# Correction: GAN-WGCNA: Calculating gene modules to identify key intermediate regulators in cocaine addiction

**DOI:** 10.1371/journal.pone.0320886

**Published:** 2025-03-21

**Authors:** Taehyeong Kim, Kyoungmin Lee, Mookyung Cheon, Wookyung Yu

There are errors in the Funding statement. The correct funding statement is as follows: This work is supported by the RandD programs of DGIST (22-CoE-BT-01), funded by the Ministry of Science and ICT of Korea. This research is supported by the KBRI Basic Research Program through the Korea Brain Research Institute, funded by the Ministry of Science and ICT (24-BR-02-04 [MC]) and the National Research Foundation of Korea (NRF) grant funded by the Korea government (MSIT) (2021R1A2C1003657 [TK,KL,MC] NRF-2023R1A2C1006248(WY)). The funders have no role in study design, data collection and analysis, decision to publish, or preparation of the manuscript.
