# Supplementary material for: GAN-WGCNA: Calculating gene modules to identify key intermediate regulators in cocaine addiction
Source: PLoS One. 2024 Oct 3;19(10):e0311164. doi: 10.1371/journal.pone.0311164 (PMC11449371; doi:10.1371/journal.pone.0311164)

S1 Fig. GAN training details a. Table of Hyper-parameters used during GAN training **b.** detailed parameters for data dimension transitions; N_{train} is the number of linear augmented samples N_{sim} is the number of simulations which has done between CN and SN, N_{gex} is number of genes which is differ among brain regions **c.** training validation through overlapping effect in tSNE space during GAN training

a.

Hyperparmeter	Value
z_dim	100
Lambda	10
L_r	0.1e-5
G	800
D	400
batch_size	32
R_threshold	0.95

b.

Region	State combination		Sample dimension			WGCNA
	Cocaine	Saline	N_{train}	N_{sim}	N_{gex}	Module_num
BLA	6	7	966	42	13558	121
VTA	6	6	644	36	13317	96
CPU	8	8	1195	64	13022	91
HIP	8	7	1030	56	13176	136
PFC	8	7	1140	56	13092	89
NAC	8	8	1048	64	13232	132

c.

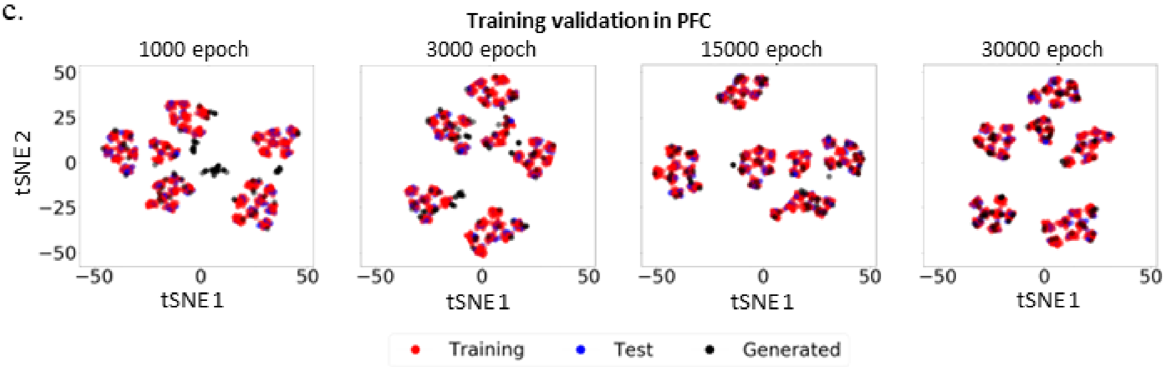

Supplement: S1 Fig — GAN training details a. Table of Hyper-parameters used during GAN training b. detailed parameters for data dimension transitions; Ntrain is the number of linear augmented samples Nsim is the number of simulations which has done between CN and SN, Ngex is number of genes which is differ among brain regions c. training validation through overlapping effect in tSNE space during GAN training. (PDF) [file pone.0311164.s001.pdf]
