# Supplementary material for: GAN-WGCNA: Calculating gene modules to identify key intermediate regulators in cocaine addiction
Source: PLoS One. 2024 Oct 3;19(10):e0311164. doi: 10.1371/journal.pone.0311164 (PMC11449371; doi:10.1371/journal.pone.0311164)

S2 Fig. Research Workflow Transitions of dataset's shape during preparing training data, model training, simulating and GAN-WGCNA a. De-scription of a used dataset (GSE110344). The tissue collection performed in 6 brain regions b. Training data preparation for GAN training. Simple 10-fold linear aug-mentation method is applied. c. GAN training framework structure d. Gene expression simulation through latent space interpolation using a trained generator model e. GAN-WGCNA using adjacency matrix from averaged gene profile and behavioral datasets from original samples which provide a spatiotemporal analysis of the tran-scriptome f. Rescued DEG used to show statistically significant gene expression profile.

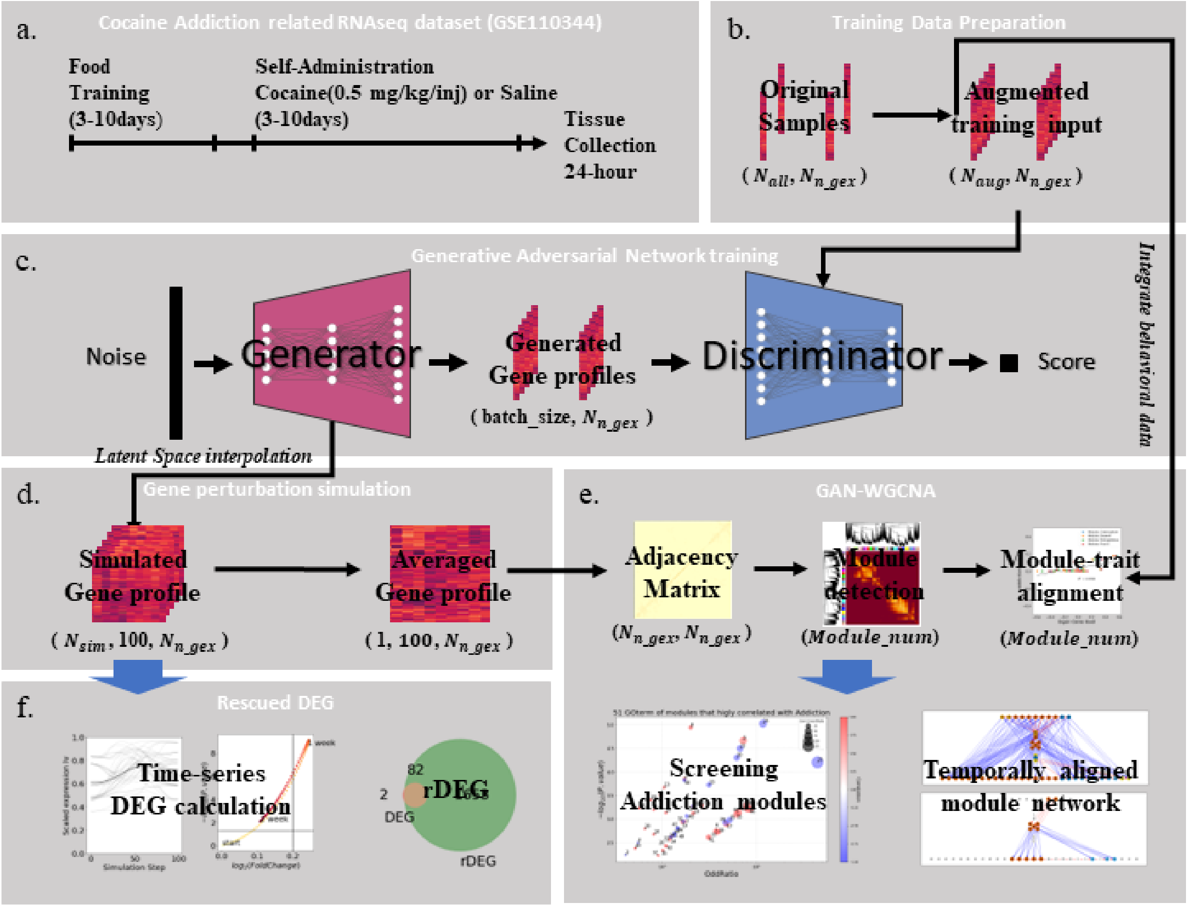

Supplement: S2 Fig — Research Workflow Transitions of dataset’s shape during preparing training data, model training, simulating and GAN-WGCNA a. De-scription of a used dataset (GSE110344). The tissue collection performed in 6 brain regions b. Training data preparation for GAN training. Simple 10-fold linear aug-mentation method is applied. c. GAN training framework structure d. Gene expression simulation through latent space interpolation using a trained generator model e. GAN-WGCNA using adjacency matrix from averaged gene profile and behavioral datasets from original samples which provide a spatiotemporal analysis of the tran-scriptome f. Rescued DEG used to show statistically significant gene expression profile. (PDF) [file pone.0311164.s002.pdf]
