# Supplementary material for: GAN-WGCNA: Calculating gene modules to identify key intermediate regulators in cocaine addiction
Source: PLoS One. 2024 Oct 3;19(10):e0311164. doi: 10.1371/journal.pone.0311164 (PMC11449371; doi:10.1371/journal.pone.0311164)

S3 Fig. Creb family genes expression profile Creb gene family expression pattern shows spatiotemporal differences which enables detailed interpretation of cocaine addiction and sophisticated module detection in WGCNA

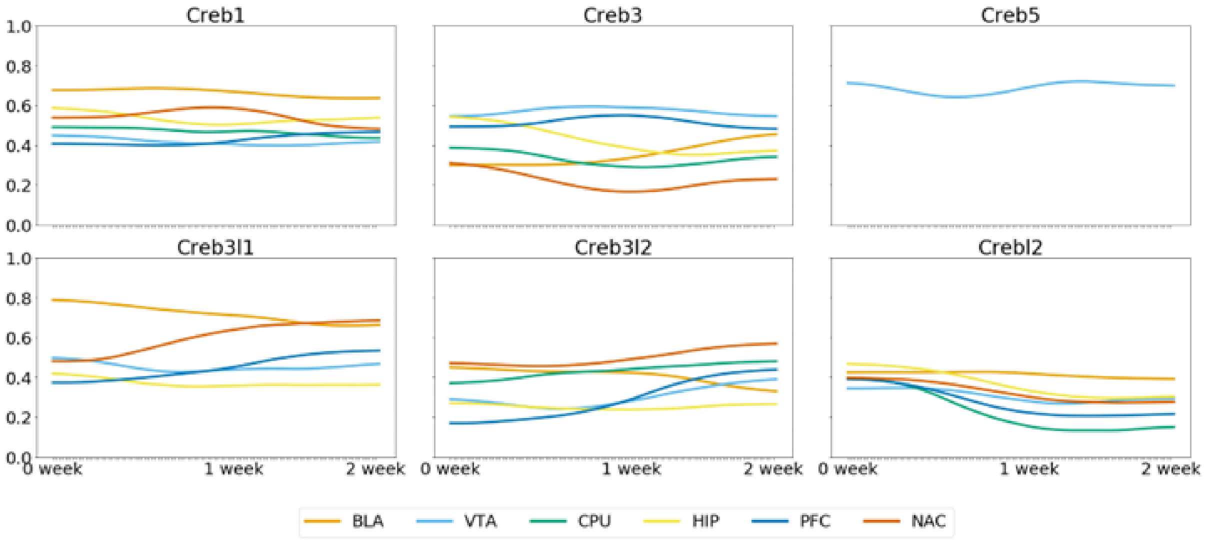

Supplement: S3 Fig — (PDF) [file pone.0311164.s003.pdf]
