# Supplementary material for: GAN-WGCNA: Calculating gene modules to identify key intermediate regulators in cocaine addiction
Source: PLoS One. 2024 Oct 3;19(10):e0311164. doi: 10.1371/journal.pone.0311164 (PMC11449371; doi:10.1371/journal.pone.0311164)

S4 Fig. WGCNA a. Selection of Soft threshold. Note that couldn't meet conventional criteria in NAc region **b.** Module was detected after calculation of Topological overlapped Matrix and Dynamic Tree Cut method which is basically cut-off dendrogram on specific height and conditions.

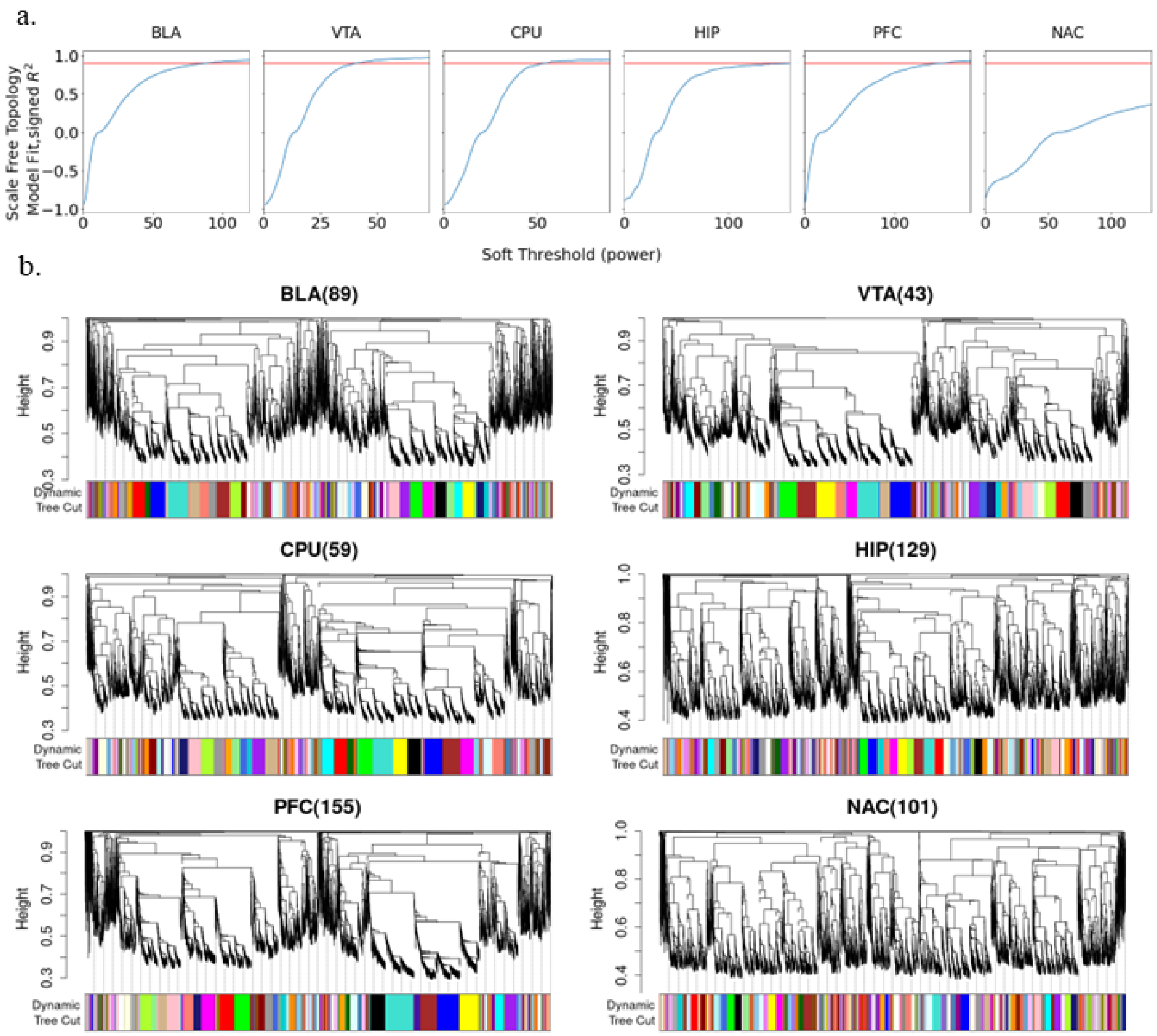

Supplement: S4 Fig — WGCNA a. Selection of Soft threshold. Note that couldn’t meet conventional criteria in NAc region b. Module was detected after calculation of Topological overlapped Matrix and Dynamic Tree Cut method which is basically cut-off dendrogram on specific height and conditions. (PDF) [file pone.0311164.s004.pdf]
