# Supplementary material for: GAN-WGCNA: Calculating gene modules to identify key intermediate regulators in cocaine addiction
Source: PLoS One. 2024 Oct 3;19(10):e0311164. doi: 10.1371/journal.pone.0311164 (PMC11449371; doi:10.1371/journal.pone.0311164)

S5 Fig. Eigen-gene profiles of dominant four selected modules in order of the addition index of original samples The eigen-gene expression profile, which is calculated in generated dataset, gives us an opportunity of calculating correlation to the additive behavior in temporal aspect i.e. addiction progress.

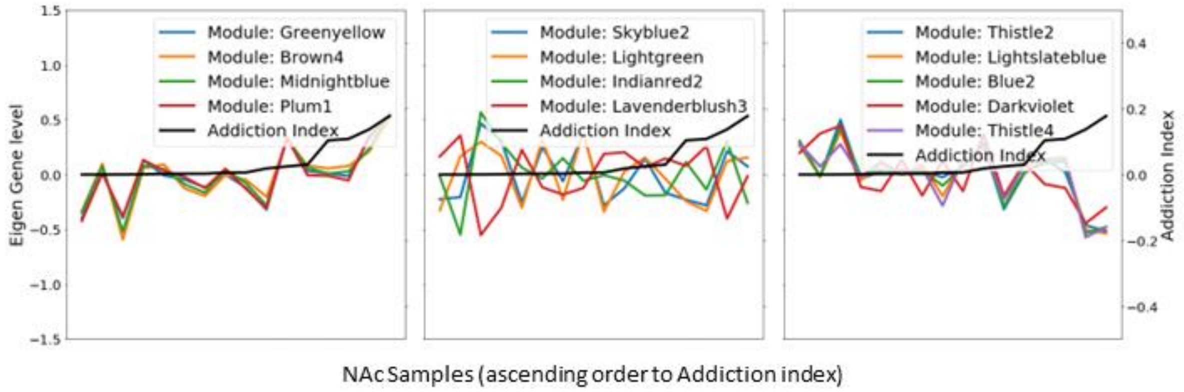

Supplement: S5 Fig — (PDF) [file pone.0311164.s005.pdf]
