# Supplementary material for: GAN-WGCNA: Calculating gene modules to identify key intermediate regulators in cocaine addiction
Source: PLoS One. 2024 Oct 3;19(10):e0311164. doi: 10.1371/journal.pone.0311164 (PMC11449371; doi:10.1371/journal.pone.0311164)

S6 Fig. Temporally aligned module network (Full) Network visualization to show full connection including day0 and day14 connection.

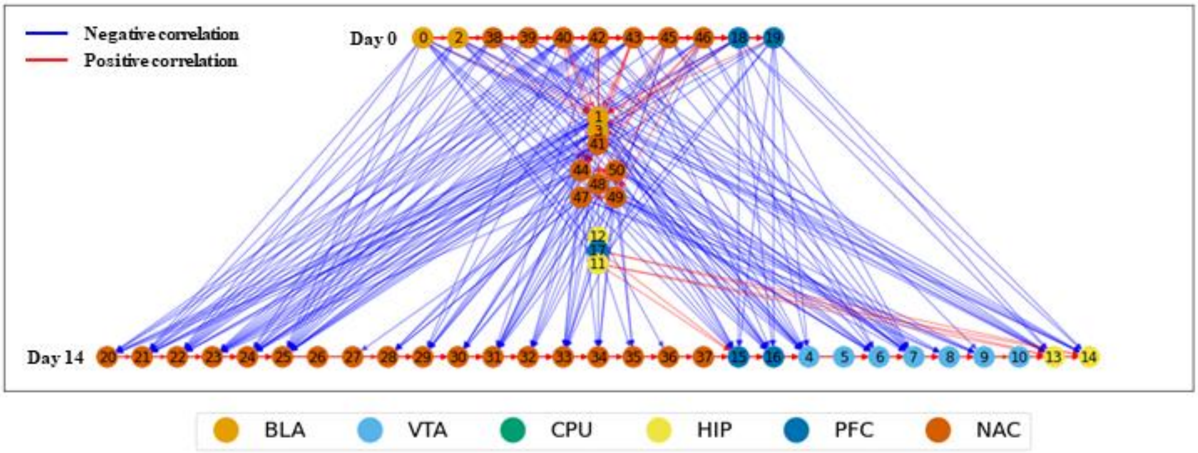

Supplement: S6 Fig — (PDF) [file pone.0311164.s006.pdf]
