# Supplementary material for: GAN-WGCNA: Calculating gene modules to identify key intermediate regulators in cocaine addiction
Source: PLoS One. 2024 Oct 3;19(10):e0311164. doi: 10.1371/journal.pone.0311164 (PMC11449371; doi:10.1371/journal.pone.0311164)

S7 Fig. Clustering of correlation matrix to distinguish correlation patterns in modules Specific correlation pattern was founded in Early-Mid-Late stage of NAc region

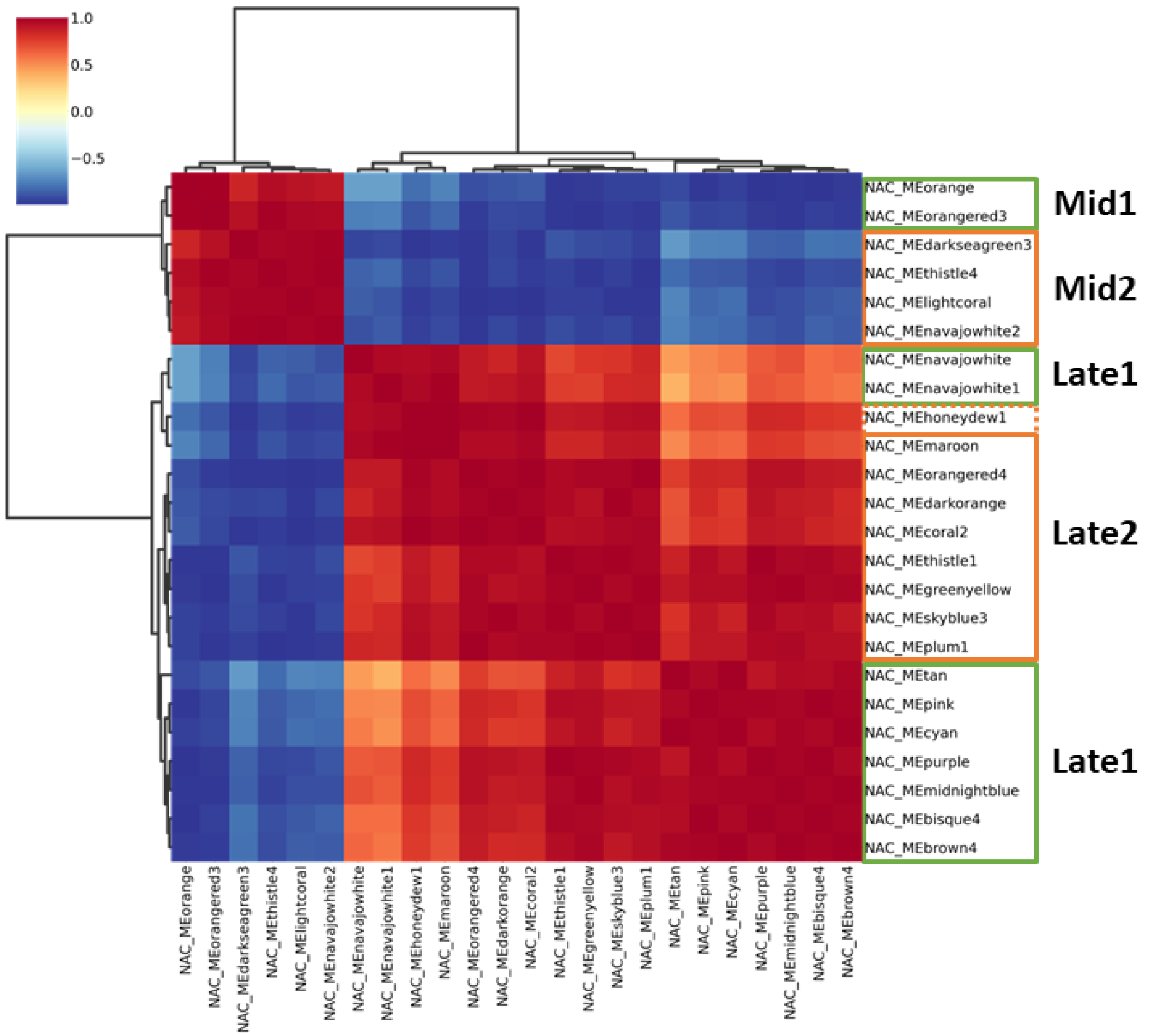

Supplement: S7 Fig — (PDF) [file pone.0311164.s007.pdf]
