# Supplementary material for: GAN-WGCNA: Calculating gene modules to identify key intermediate regulators in cocaine addiction
Source: PLoS One. 2024 Oct 3;19(10):e0311164. doi: 10.1371/journal.pone.0311164 (PMC11449371; doi:10.1371/journal.pone.0311164)
Supplement: S8 Fig — GO results a. GO result of Mid1, Mid2, Late1, Late2 groups in NAc. b. Comparison of GO DAG between Late1 and Late2. (PDF) [file pone.0311164.s008.pdf]

S8 Fig. GO results a. GO result of Mid1, Mid2, Late1, Late2 groups in NAc. **b.** Comparison of GO DAG between Late1 and Late2

a.

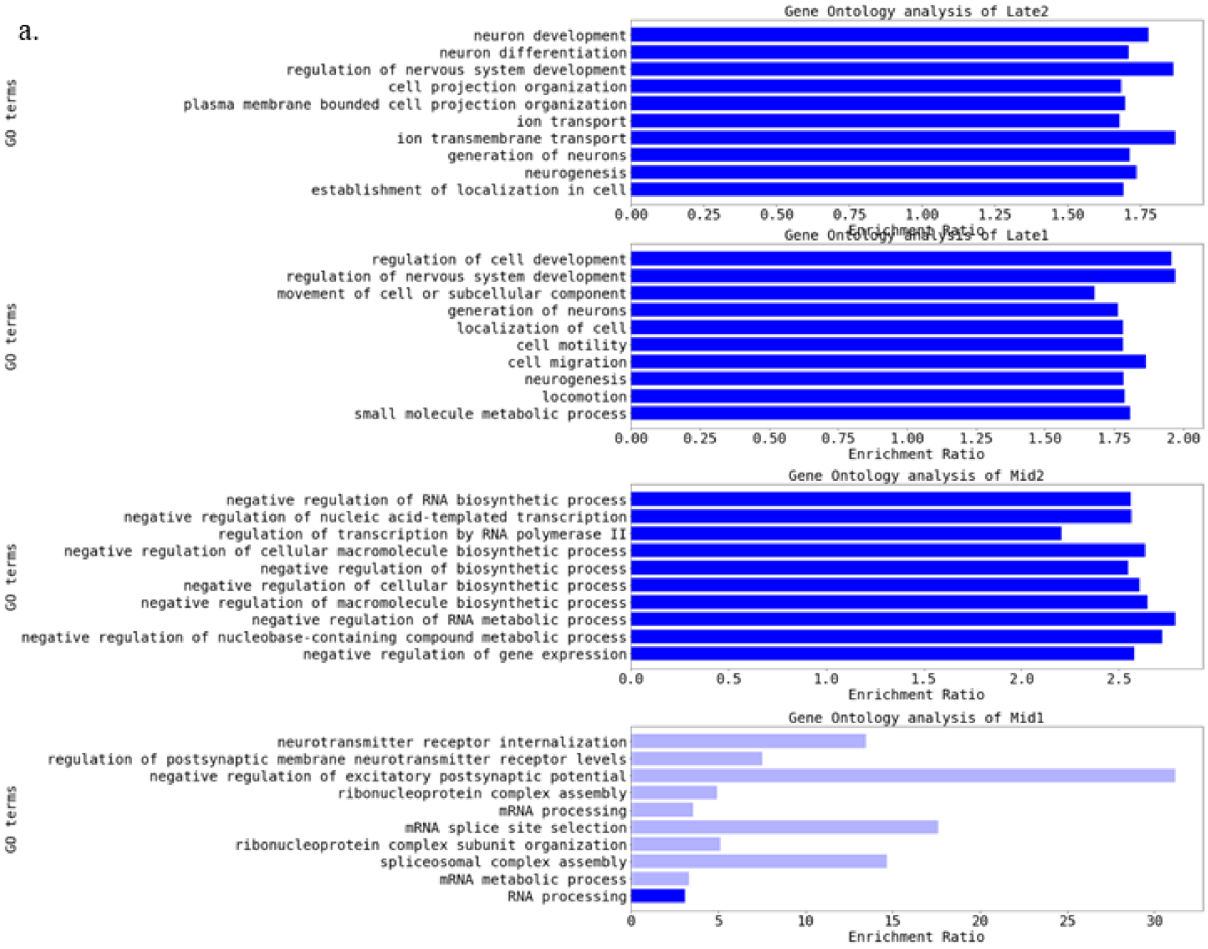

b.

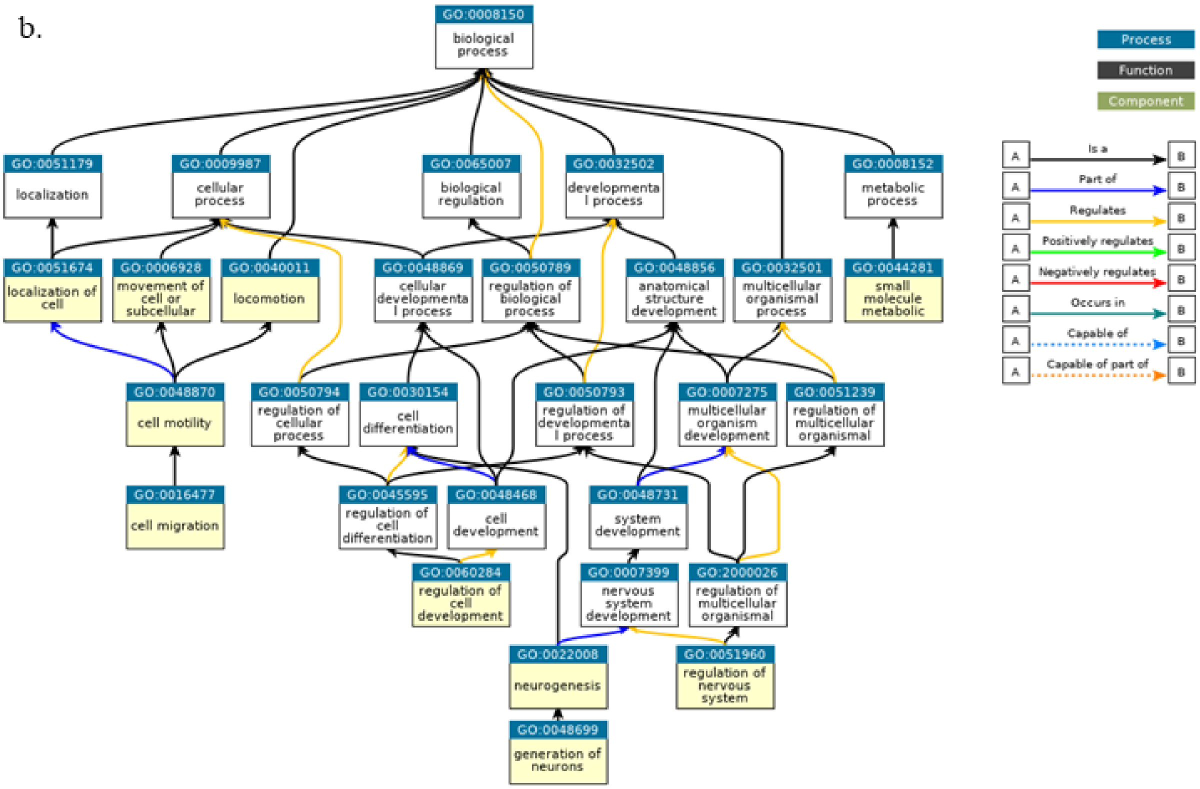

QuickGO - https://www.ebi.ac.uk/QuickGO

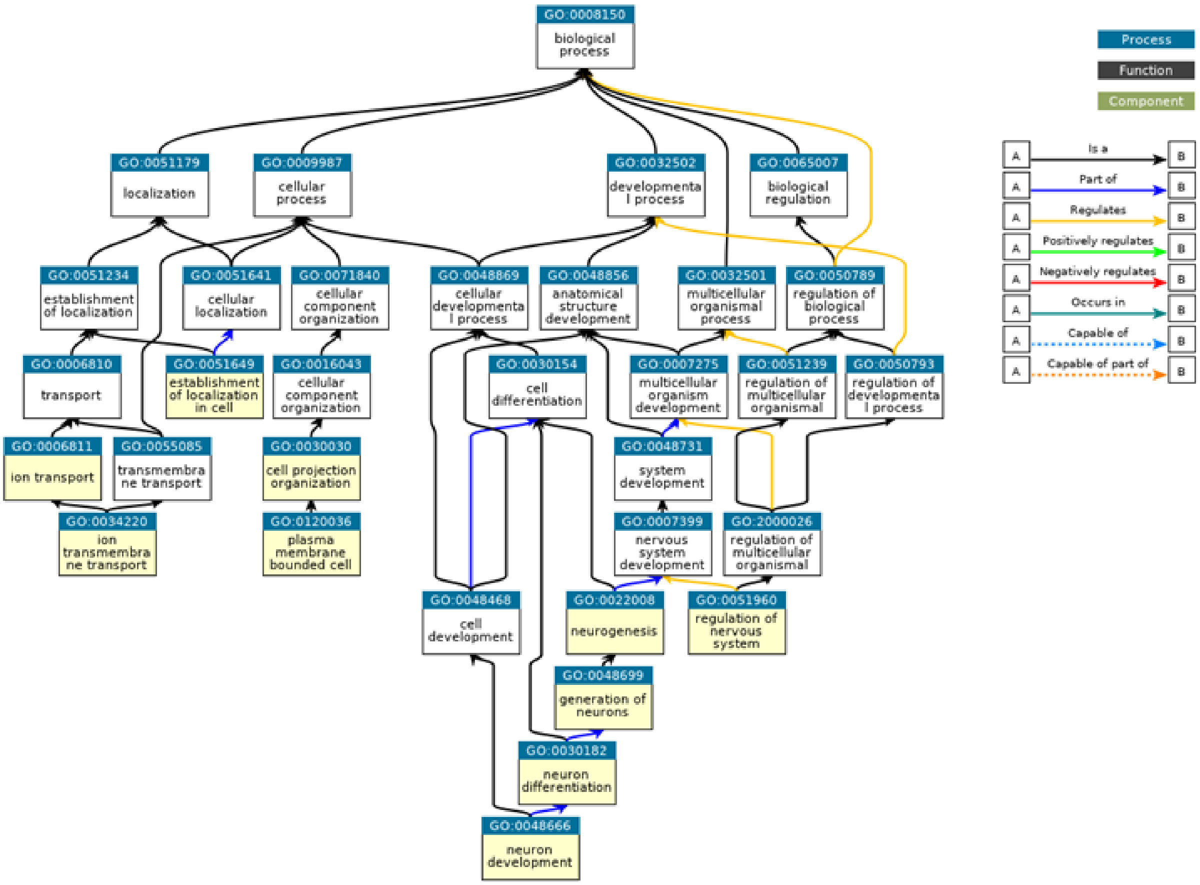

QuickGO - https://www.ebi.ac.uk/QuickGO
