# Supplementary material for: GAN-WGCNA: Calculating gene modules to identify key intermediate regulators in cocaine addiction
Source: PLoS One. 2024 Oct 3;19(10):e0311164. doi: 10.1371/journal.pone.0311164 (PMC11449371; doi:10.1371/journal.pone.0311164)

**S9 Fig. rDEG visualization of Alcam, Celf4 and Cdh11 a.** Visualized examples are intermediate example that are possible DEG in intermediate stages **b.** rDEG-filtered GO result shows detailed term comparing to the non-filtered result **c.** Comparison between DEG and rDEG shows rDEG is an extended version of DEG.

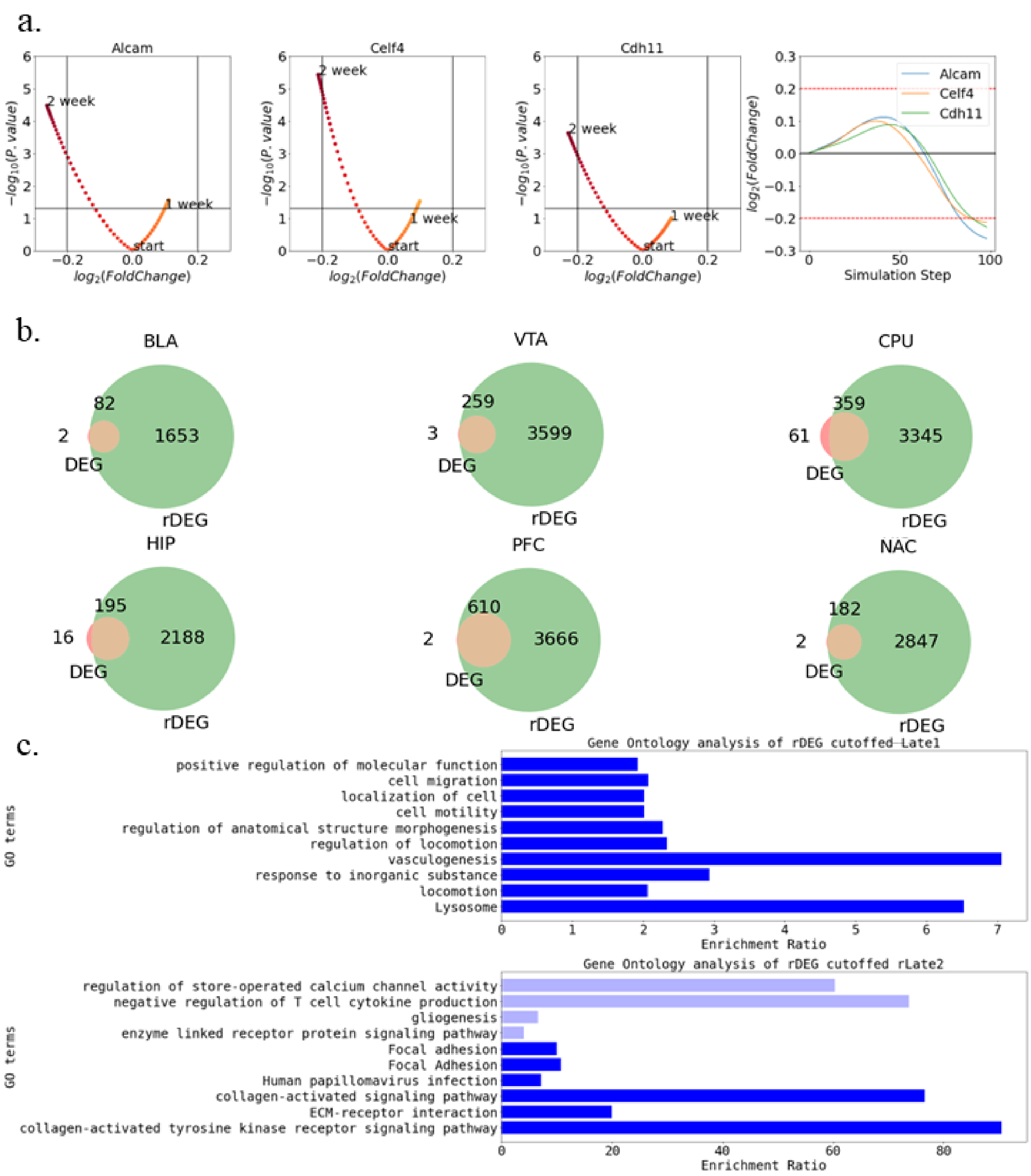

Supplement: S9 Fig — rDEG visualization of Alcam, Celf4 and Cdh11 a. Visualized examples are intermediate example that are possible DEG in intermediate stages b. rDEG-filtered GO result shows detailed term comparing to the non-filtered result c. Comparison between DEG and rDEG shows rDEG is an extended version of DEG. (PDF) [file pone.0311164.s009.pdf]
