# Supplementary material for: GAN-WGCNA: Calculating gene modules to identify key intermediate regulators in cocaine addiction
Source: PLoS One. 2024 Oct 3;19(10):e0311164. doi: 10.1371/journal.pone.0311164 (PMC11449371; doi:10.1371/journal.pone.0311164)

S10 Fig. Spatiotemporal expression patterns of behavioral related modules Three visualized examples for each behavior related gene modules in different brain regions – module 48 in NAc, module 16 in PFC, and module 10 in VTA. Six representative genes were selected for visualization.

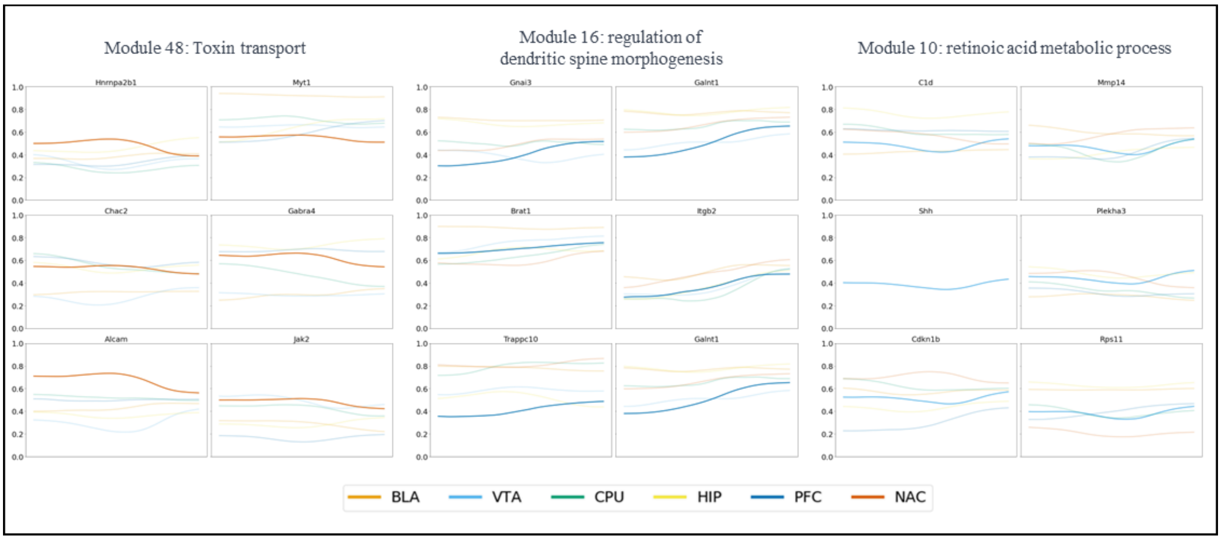

Supplement: S10 Fig — Six representative genes were selected for visualization. (PDF) [file pone.0311164.s010.pdf]
