# Supplementary material for: GAN-WGCNA: Calculating gene modules to identify key intermediate regulators in cocaine addiction
Source: PLoS One. 2024 Oct 3;19(10):e0311164. doi: 10.1371/journal.pone.0311164 (PMC11449371; doi:10.1371/journal.pone.0311164)

S11 Fig. Comparison between WGCNA and GAN-WGCNA a. Differences of WGCNA and GAN-WGCNA in three aspects **b.** Distinguishing features of GAN-WGCNA in identifying and computing eigengenes across different samples.

a.

	Input	Module	Module correlation
**WGCNA**	Gene expression level of real samples	Genes are grouped based on their expression level among samples → generally co-expressed genes	Correlation implies proportionality between module expression level and trait level
**GAN-WGCNA**	Times-series expression level of generated samples	Genes are grouped based on their time-series expression level → co-expressed genes during transition	Correlation implies similarity of transition pattern between module expression level and trait level

b.

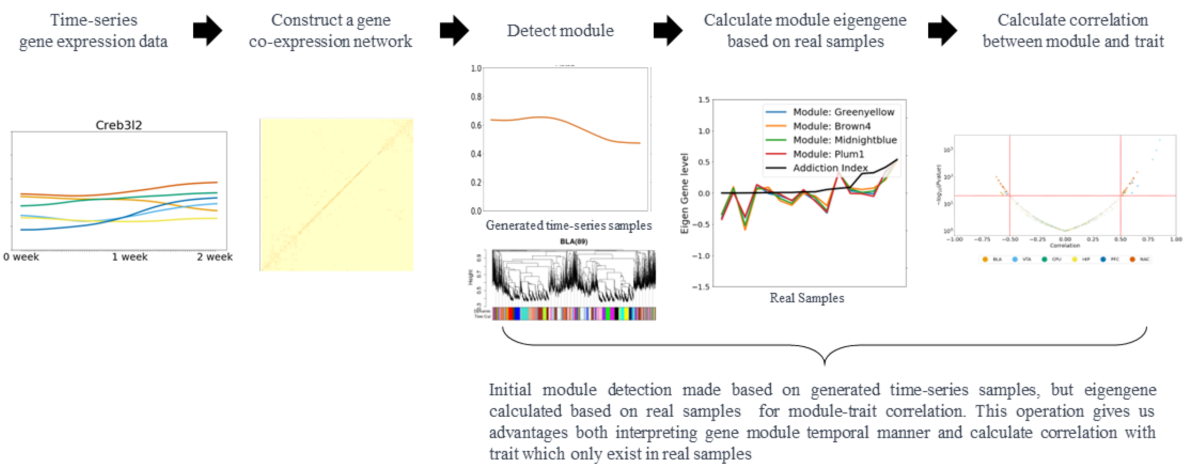

Supplement: S11 Fig — Comparison between WGCNA and GAN-WGCNA a. Differences of WGCNA and GAN-WGCNA in three aspects b. Distinguishing features of GAN-WGCNA in identifying and computing eigengenes across different samples. (PDF) [file pone.0311164.s011.pdf]
