# Supplementary material for: GAN-WGCNA: Calculating gene modules to identify key intermediate regulators in cocaine addiction
Source: PLoS One. 2024 Oct 3;19(10):e0311164. doi: 10.1371/journal.pone.0311164 (PMC11449371; doi:10.1371/journal.pone.0311164)
Supplement: S1 Table — Corr and P.val calculated from WGCNA module-trait alignment and G.Pv(Pvalue) and OR(OddRatio) and ER(EnrichmentRatio) calculated from GO analysis. (PDF) [file pone.0311164.s012.pdf]

S1 Table. GO table of gene module Modules' GO terms and correlation calculation results. Corr and P.val calculated from WGCNA module-trait alignment and G.Pv(Pvalue) and OR(OddRatio) and ER(EnrichmentRatio) calculated from GO analysis.

id	Region	GOterm	Corr	P.val	G_P.val	OR	ER
0	**BLA**	positive regulation of cardiac muscle hypertrophy	-0.57983	0.037789	0.001561	14.6102	12.97107
1	**BLA**	macrophage migration	-0.56401	0.044673	0.000867	18.28241	15.81349
2	**BLA**	glycosylceramide metabolic process	-0.56638	0.043592	0.002246	13.53917	11.16246
3	**BLA**	maternal process involved in female pregnancy	-0.57867	0.038266	0.000149	17.23761	14.65074
4	**VTA**	cellular component maintenance	0.822947	0.00101	0.004186	5.162849	4.665905
5	**VTA**	positive regulation of cilium assembly	0.652663	0.021409	0.002431	31.1746	27.10069
6	**VTA**	microglial cell activation	0.789909	0.002238	0.003703	10.93125	9.476927
7	**VTA**	myofibril assembly	0.853435	0.000414	0.000585	5.697147	4.679971
8	**VTA**	regulation of stem cell division	0.601669	0.038478	0.00072	64.73626	48.73663
9	**VTA**	response to pain	0.654322	0.02097	0.00039	7.574274	5.933862
10	**VTA**	retinoic acid metabolic process	0.601375	0.038598	0.000614	85.11594	50.39574
11	**HIP**	peptidyl-lysine hydroxylation	0.547215	0.034752	0.000512	79.28571	57.41176
12	**HIP**	negative regulation of reactive oxygen species biosynthetic process	0.524764	0.044597	0.005895	9.054945	8.077241
13	**HIP**	odontogenesis of dentin-containing tooth	0.514837	0.049553	0.001597	6.525404	5.822231
14	**HIP**	positive regulation of growth hormone secretion	0.543617	0.036209	0.001855	39.52041	30.26357
15	**PFC**	regulation of cilium movement	0.607209	0.016366	0.000514	26.30275	17.63952
16	**PFC**	regulation of dendritic spine morphogenesis	0.539085	0.038107	0.0003	9.55731	8.40158
17	**PFC**	regulation of dendritic spine morphogenesis	0.516371	0.048761	0.000141	17.26677	14.99359
18	**PFC**	mitochondrial respiratory chain complex I assembly	-0.54393	0.036082	0.005756	4.080888	3.624483
19	**PFC**	regulation of mitotic sister chromatid segregation	-0.52799	0.043069	0.003182	5.526647	4.972364
20	**NAC**	regulation of ventricular cardiac muscle cell membrane repolarization	0.542089	0.030067	2.16E-05	72.7875	52.97285
21	**NAC**	regulation of T cell differentiation in thymus	0.553438	0.026152	0.001812	37.32051	31.21867
22	**NAC**	negative regulation of blood coagulation	0.540829	0.030528	0.001125	17.24627	14.24209
23	**NAC**	peptidyl-cysteine modification	0.616589	0.010963	0.000628	8.161451	7.147131
24	**NAC**	positive regulation of epithelial cell differentiation	0.616804	0.010927	1.14E-05	20.09722	16.5587
25	**NAC**	copper ion homeostasis	0.586856	0.016858	0.000745	21.32407	15.99317
26	**NAC**	negative regulation of lipid storage	0.528013	0.035526	0.001872	38.000	30.40779
27	**NAC**	mesenchyme morphogenesis	0.515275	0.041083	0.000544	21.46367	18.60222
28	**NAC**	regulation of ubiquitin-protein transferase activity	0.529478	0.034925	0.000212	10.4444	9.049938
29	**NAC**	regulation of dendritic spine morphogenesis	0.583465	0.017663	0.002364	12.46989	11.25673
30	**NAC**	SREBP signaling pathway	0.526621	0.036104	0.000792	61.60847	46.45635
31	**NAC**	type B pancreatic cell proliferation	0.648863	0.00654	0.004646	10.13845	8.710565
32	**NAC**	monoubiquitinated protein deubiquitination	0.60127	0.013753	0.00127	48.15321	36.64163
33	**NAC**	release of cytochrome c from mitochondria	0.613588	0.011471	0.00211	8.131217	7.323741
34	**NAC**	regulation of antigen receptor-mediated signaling pathway	0.584579	0.017395	0.001154	16.34225	14.38206
35	**NAC**	cochlea development	0.53916	0.031146	0.002733	12.02391	10.62341
36	**NAC**	activation of JNKK activity	0.522081	0.038038	0.000597	79.64384	52.03111
37	**NAC**	chaperone-mediated protein folding	0.55615	0.025277	0.000731	10.94981	9.755833
38	**NAC**	adhesion of symbiont to host	-0.56771	0.021797	3.18E-05	67.31467	45.61169

39	**NAC**	miRNA loading onto RISC involved in gene silencing by miRNA	-0.5656	0.022401	9.40E-06	117.8311	65.15955
40	**NAC**	deoxyribonucleoside metabolic process	-0.58576	0.017115	6.25E-05	448.1538	144.5309
41	**NAC**	negative regulation of smooth muscle cell proliferation	-0.56081	0.023827	0.001579	14.57394	12.91213
42	**NAC**	neuroepithelial cell differentiation	-0.60236	0.013537	0.0024	12.39879	11.1993
43	**NAC**	glycosyl compound biosynthetic process	-0.62244	0.010024	0.002003	13.51986	11.82525
44	**NAC**	regulation of mRNA polyadenylation	-0.54563	0.028799	0.001162	47.18219	39.02333
45	**NAC**	positive regulation of inositol phosphate biosynthetic process	-0.55606	0.025304	4.99E-05	62.07321	38.175
46	**NAC**	polysaccharide biosynthetic process	-0.60404	0.013211	0.000533	21.52407	18.76122
47	**NAC**	negative regulation of cation channel activity	-0.52888	0.035168	0.001111	16.81044	14.48289
48	**NAC**	toxin transport	-0.58176	0.018079	0.000188	31.45135	26.60682
49	**NAC**	heparan sulfate proteoglycan biosynthetic process	-0.51418	0.041588	0.002094	13.50877	11.56818
50	**NAC**	chemokine secretion	-0.53132	0.034181	0.001058	51.96875	40.36897
