# Supplementary material for: GAN-WGCNA: Calculating gene modules to identify key intermediate regulators in cocaine addiction
Source: PLoS One. 2024 Oct 3;19(10):e0311164. doi: 10.1371/journal.pone.0311164 (PMC11449371; doi:10.1371/journal.pone.0311164)
Supplement: S2 Table — (PDF) [file pone.0311164.s013.pdf]

S2 Table. Genes in intermediate modules notable genes in intermediate modules (41, 44, 47, 48, 49, 50) and its references.

Id	Gene	Reference journal	Links	Title
41	Efnb2	Curr Neuropharmacol	https://dx.doi.org/10.2174%2F1570159X16666180416093717	Possible Cytoeskeletal adapters; Possible protein-linkers mediating adenosine and metabotropic glutamate receptors crosstalk at different levels, according to their possible roles in relation to GPCR function
41	Nampt	Int J Physiol Pathophysiol Pharmacol.	https://pubmed.ncbi.nlm.nih.gov/31993108	Nicotinamide phosphoribosyltransferase contributes to cocaine addiction through sirtuin 1
41	Zc3h14	J biol Chem	10.1074/jbc.RA120.015389	An endogenous PI3K interactome promoting astrocyte-mediated neuroprotection identifies a novel association with RNA-binding protein ZC3H14
41	Zc3h13	Cell Death & Disease	https://doi.org/10.1038/s41419-020-03143-z	The role of m6A modification in physiology and disease; m6A to addcition
41	Celf4	PLoS Genet	10.1371/journal.pgen.1003067	CELF4 regulates translation and local abundance of a vast set of mRNAs, including genes associated with regulation of synaptic function
		Genes, Brain and Behavior	https://doi.org/10.1111/gbb.12273	RNA-binding proteins, neural development and the addictions
41	Auts2	Drug and Alcohol Dependence	10.1016/j.drugalcdep.2012.08.029	Genetic analysis of AUTS2 as a susceptibility gene of heroin dependence
41	St6galnac5	Molecular Psychiatry	https://doi.org/10.1038/mp.2014.159	Transcriptome organization for chronic alcohol abuse in human brain
41	Cmc1	Alcohol Clin Exp Res	https://doi.org/10.1038/mp.2014.159	Genome-wide association study of alcohol dependence implicates a region on chromosome 11
41	Phtf2	Alcohol	10.1016/j.alcohol.2016.08.008	Analyses of differentially expressed genes after exposure to acute stress, acute ethanol, or a combination of both in mice
41	rDEGs does not have Ref in this table: Plekha3, Qrich1, Ypel2, Smc3, Dcaf6, Mrps31, Upf3b, Gpatch2, Dnttip2, Cir1, Slitrk6, Erich1, Rpl23a, Tsga10, Zfp442, Gm24111			
44	Jakmip2	Cancer Research	10.1158/0008-5472.CAN-20-0057 : https://jamanetwork.com/ on 12/09/2021	A genome-wide pooled shRNA screen identifies PPP2R2A as a predictive biomarker Genome-wide Association Study of Alcohol Dependencefor the response to ATR and CHK1 inhibitors
44	Pcmtd2	Nature genomic medicine	https://doi.org/10.1038/s41525-019-0098-3	A large data resource of genomic copy number variation across neurodevelopmental disorders

44	Usp1	JNCI	?https://doi.org/10.1093/jnci/djx059	Drugging the Cancers Addicted to DNA Repair?
44	rDEGs does not have Ref in this table: Prkar2b, Ppp2r2a, Tgs1, Kdm4c, Scaper, Ythdc1, Anapc10, Bcl11b, Zfp961, Epc2			
47	Fmr1	Heliyon	10.1016/j.heliyon.2020.e05270	Decreased?FMR1?mRNA levels found in men with substance use disorders
47	Naa50	Heliyon	10.1016/j.neuroscience.2014.08.041	Understanding the addiction cycle: a complex biology with distinct contributions of genotype vs. sex at each stage
47	Lypla1	Probe Reports from the NIH Molecular Libraries Program [Internet]. Bethesda (MD): National Center for Biotechnology Information	https://www.ncbi.nlm.nih.gov/books/NBK143552/	Optimization and characterization of triazole urea inhibitors for abhydrolase domain containing protein 6 (ABHD6)
47	Flrt3	Structure	10.1016/j.str.2015.06.022	Structural and Mechanistic Insights into the Latrophilin3-FLRT3 Complex that Mediates Glutamatergic Synapse Development
47	Nyap2	Oncogene	10.1038/s41388-019-1136-4	The Mechanism of Cancer Drug Addiction in ALK-Positive T-Cell Lymphoma
47	Gulp1	Alcohol	?10.1016/j.alcohol.2016.08.008	Analyses of differentially expressed genes after exposure to acute stress, acute ethanol, or a combination of both in mice; behavioral phenotype
47	rDEGs does not have Ref in this table: Cyria, Gt(ROSA)26Sor			
48	Alcam	J neuro	https://doi.org/10.1523/JNEUROSCI.0278-19.2019	Axonal Growth of Midbrain Dopamine Neurons is Modulated by the Cell Adhesion Molecule ALCAM Through?Trans-Heterophilic Interactions with L1cam, Chl1, and Semaphorins
48	Pja2	PLoS One	10.1371/journal.pone.0037999	Amygdala 14-3-3?? as a Novel Modulator of Escalating Alcohol Intake in Mice
48	Spock3	Scientific Reports	https://doi.org/10.1038/s41598-017-10207-2	Association of the?PLCB1?gene with drug dependence
48	Macrod2	Psychiatr Genet.	10.1097/YPG.0000000000000052	MACROD2?gene associated with autistic-like traits in a general population sample
49	rDEGs does not have Ref in this table: Meis2, Cdh11, Mphosph8			

50	Lnpk	Sci Rep.	?10.1038/s41598-021-93085-z	Machine learning compensates fold-change method and highlights oxidative phosphorylation in the brain transcriptome of Alzheimer's disease
50	Orc4	AIMS Molecular Science	10.3934/molsci.2021016	Genetic variation in alcoholism and opioid addiction susceptibility and treatment: a pharmacogenomic approach
50	Zranb2	Sci Rep	10.1038/srep36993	mRNA changes in nucleus accumbens related to methamphetamine addiction in mice
50	Eif1a	EMBO rep	https://doi.org/10.15252/embr.201642195	The integrated stress response
		The neuroscientist	https://doi.org/10.1177/1073858419853236	Protein Translation and Psychiatric Disorders
50	Mbtd1	Genes Brain Behav	?10.1111/j.1601-183X.2009.00525.x	A major QTL on chromosome 11 influences psychostimulant and opioid sensitivity in mice
50	rDEGs does not have Ref in this table: Ggnbp2, Zmynd11, Spopl, Tsc22d2, Plppr4, Pcnp, Gm14148			
