# Supplementary material for: GAN-WGCNA: Calculating gene modules to identify key intermediate regulators in cocaine addiction
Source: PLoS One. 2024 Oct 3;19(10):e0311164. doi: 10.1371/journal.pone.0311164 (PMC11449371; doi:10.1371/journal.pone.0311164)
Supplement: S1 File — (PDF) [file pone.0311164.s014.pdf]

S1 Note. C57BL/6J cocaine self-administration dataset

We started our analysis with male C57BL/6J mouse bulk-mRNA-seq count data, which were provided by the authors upon request. The dataset consists of six brain region transcriptomes from 230 samples (3–6 samp les per region) and their behavioral scores.

In the previous study, the authors collected transcriptome and behavioral data based on the following experimental design: 3–10 d of food training and 10–15 d of cocaine self-administration, and intraperitoneal (IP) injection 30d after self-administration. Therefore, there are six conditions, which are a combination of self-administration (SA) and challenge (IP injection after withdrawal). In SA, there are two conditions: cocaine is self-administrated (cocaine, C; saline, S). In the challenge, there are three conditions: IP injection of cocaine or saline after 30 d of withdrawal and no challenge treatment, and the mice are euthanized 24 h after SA (Challenge; C or S, None; N). Three contextual DEG analyses were performed in a previous study: cocaine addiction (DEG from SN to CN), cocaine re-exposure (DEG from CC to CN), and acute cocaine injection (DEG from SS to SC).

The reason for choosing this dataset in this research is not only because it is well-performed bulk mRNAseq data covering six brain regions, which provides us with the opportunity to demonstrate spatiotemporal transcriptome analysis, but also because the dataset includes a behavioral trait called the addiction index(AI). AI is a composite score of several addiction-like behaviors during cocaine self-administration, three behaviors (cocaine intake, discrimination of active vs inactive lever pressing, and consummatory regulation) were selected and used to generate AI using exploratory factor analysis method. AI has been shown to have a distinct correlation with cocaine self-administration in a previous study and we utilize for filtering significant gene modules related to addictive behavior.
