# Supplementary material for: GAN-WGCNA: Calculating gene modules to identify key intermediate regulators in cocaine addiction
Source: PLoS One. 2024 Oct 3;19(10):e0311164. doi: 10.1371/journal.pone.0311164 (PMC11449371; doi:10.1371/journal.pone.0311164)
Supplement: S2 File — (PDF) [file pone.0311164.s015.pdf]

S2 Note. Voom and data preprocessing for training

From the provided count data, we calculated reads per kilobase per million mapped reads (RPKM) using edgeR [1-3] and the biomaRT library [4, 5]. We filtered out negligibly small amounts of data from the RPKM profile using the same criteria as that used in the previous study. In the previous study, genes were maintained with more than 1 RPKM in at least 80% of the samples per group. We used the RPKM cut-off approach only for unbiased systemic level analysis, which maintained a larger number of genes, more than ten thousand, compared to using approximately a thousand genes in a previous GAN study [6] which used the DEG cut-off in their training data.

Then, we normalized the RPKM data using the voom library from R[7]. Voom normalized count data to log2-counts per million (logCPM) based on its treatment factors, batch effects, or numerical covariates [7]. In this case, there were six treatment states (CC, CS, SC, SS, SN, and CN) for each brain region.

$$Sample_{aug} = S_i + (1 - \mathrm{x})S_j \ \ (where\ \mathrm{x\ is\ 0.1}\ to\ 0.9)$$

For generative model training, we augmented our dataset through 10-fold linear interpolation, which was the same as that in a previous study [6]. The above equation was applied to the augmentation process, where S represents the expression value (logCPM) for each sample in the same region and treatment. They were rescaled using MinMaxScaler in the scikit-learn package [8] of Python, which normalizes the data unit into 0 to 1 values to provide more stable convergence manifolds in GAN using the leaky ReLU activation function.

References

1. Robinson MD, McCarthy DJ, Smyth GK. edgeR: a Bioconductor package for differential expression analysis of digital gene expression data. Bioinformatics. 2009;26(1):139-40. doi: 10.1093/bioinformatics/btp616.
2. McCarthy DJ, Chen Y, Smyth GK. Differential expression analysis of multifactor RNA-Seq experiments with respect to biological variation. Nucleic Acids Research. 2012;40(10):4288-97. doi: 10.1093/nar/gks042.
3. Chen Y, Lun A, Smyth G. From reads to genes to pathways: differential expression analysis of RNA-Seq experiments using Rsubread and the edgeR quasi-likelihood pipeline [version 2; peer review: 5 approved]. F1000Research. 2016;5(1438). doi: 10.12688/f1000research.8987.2.
4. Durinck S, Spellman PT, Birney E, Huber W. Mapping identifiers for the integration of genomic datasets with the R/Bioconductor package biomaRt. Nature Protocols. 2009;4(8):1184-91. doi: 10.1038/nprot.2009.97.
5. Durinck S, Moreau Y, Kasprzyk A, Davis S, De Moor B, Brazma A, et al. BioMart and Bioconductor: a powerful link between biological databases and microarray data analysis. Bioinformatics. 2005;21(16):3439-40. Epub 2005/08/06. doi: 10.1093/bioinformatics/bti525. PubMed PMID: 16082012.
6. Park J, Kim H, Kim J, Cheon M. A practical application of generative adversarial networks for RNA-seq analysis to predict the molecular progress of Alzheimer's disease. PLOS Computational Biology. 2020;16(7):e1008099. doi: 10.1371/journal.pcbi.1008099.
7. Law CW, Chen Y, Shi W, Smyth GK. voom: precision weights unlock linear model analysis tools for RNA-seq read counts. Genome Biology. 2014;15(2):R29. doi: 10.1186/gb-2014-15-2-r29.
8. Pedregosa F, Varoquaux G, Gramfort A, Michel V, Thirion B, Grisel O, et al. Scikit-learn: Machine Learning in Python. J Mach Learn Res. 2011;12(null):2825–30.
