# Supplementary material for: GAN-WGCNA: Calculating gene modules to identify key intermediate regulators in cocaine addiction
Source: PLoS One. 2024 Oct 3;19(10):e0311164. doi: 10.1371/journal.pone.0311164 (PMC11449371; doi:10.1371/journal.pone.0311164)
Supplement: S3 File — (PDF) [file pone.0311164.s016.pdf]

S3 Note. Weighted gene co-expression network analysis

First, the adjacency matrix based on the averaged gene expression profile data was calculated using adjacency function with type “signed” option for further analysis. In WGCNA, biological networks are considered to be scale-free topological networks. Based on this premise, users of the WGCNA should choose a threshold parameter to calculate the adjacency matrix for the following analysis. To support this process, the pickSoftThreshold function was used in the WGCNA library. Using the pickSoftThreshold function, we selected the thresholds for each brain region in the transcriptome which were 155 for PFC, 101 for NAc, 43 for VTA, 89 for BLA and 59 for BStr and 129 for HIP. We note that the threshold for NAc was selected as the power value at which $signed\ R^2$ value started to saturate.

Next, gene clustering was performed using the topological overlap matrix (TOM)-based dissimilarity. The TOMsimilarity function was used to calculate the similarity between genes with the “signed” option for TOMType. The TOMType option was also "signed”. The TOMsimilarity function takes the adjacency matrix and the threshold value, then returns the tree structure of the genes. Large branches in the tree structure imply gene modules that are co-expressed in the gene expression data.

Finally, from the calculated similarity, which is represented as a dendrogram, the gene can be assigned to gene modules based on various hierarchical clustering methods, which can be understood as branch-cutting methods. We used the cuttreeDynamic function [1] to assign consensus gene modules. cuttreeDynamic is a novel branch cutting method that attempts to overcome the disadvantages of classical clustering methods, such as a constant height cutoff. It assigns genes to modules through adaptive and iterative processes until the number of clusters becomes stable, which provides several advantages, including the capacity to handle nested clusters and detect outliers. We used default parameters and options except minModuleSize which was as 30.

After WGCNA, gene modules are sorted into specific time points based on a simple premise: the importance of the gene module is maximized when their eigengene expression level is high, thus interpreting the temporal importance and correlation of gene modules. Because of this gene module alignment process, we were able to construct a spatiotemporal gene expression map of cocaine addiction in six brain regions.

Furthermore, using the correlation protocol in WGCNA, which calculates the Pearson correlation between the module’s eigengene profile and feature data, the correlation between the gene module and addictive behavior phenotype (addiction index) was calculated to screen significant gene modules among gene modules. Inspection of the correlation in a time-series manner is only possible by combining gene modules that are assigned to the generated time-series gene expression data with the original gene expression data, which have a lower sample number but are paired with behavioral data. The correlation threshold used for visualization was 0.95.

References

1. Langfelder P, Zhang B, Horvath S. Defining clusters from a hierarchical cluster tree: the Dynamic Tree Cut package for R. Bioinformatics. 2008;24(5):719-20. Epub 2007/11/21. doi: 10.1093/bioinformatics/btm563. PubMed PMID: 18024473.
