# Supplementary material for: GAN-WGCNA: Calculating gene modules to identify key intermediate regulators in cocaine addiction
Source: PLoS One. 2024 Oct 3;19(10):e0311164. doi: 10.1371/journal.pone.0311164 (PMC11449371; doi:10.1371/journal.pone.0311164)
Supplement: S4 File — (PDF) [file pone.0311164.s017.pdf]

S4 Note. Spatiotemporal aspects of cocaine addiction and implications based on results

Cocaine addiction alters the brain's transcriptome, particularly in the reward circuitry, which consists of several brain regions, including the nucleus accumbens (NAc), prefrontal cortex (PFC), and ventral tegmental area (VTA). Indeed, these spatiotemporal alterations can be characterized by drug-seeking behavior, intense craving, and compulsive drug use, with each brain region contributing to these behaviors.

The NAc, for example, is known to play a crucial role in regulating reward and motivation, and cocaine-induced alterations in gene expression within this region have been implicated in the development of drug-seeking behavior and addiction[1]. In our study, module 41, which includes Celf4, and module 48, which includes Alcam, were highlighted in the NAc.

Considering that both Alcam- and Celf4-included modules have a correlation with the addiction index, their primary gene function in cocaine addiction and how they contribute addictive behavior can be explained in the context of each gene. Alcam is involved in the regulation of cell adhesion, migration, and differentiation, and it has been implicated in the synaptic plasticity of the NAc. Additionally, previous studies show Alcam is a possible neuroinflammation modulator[2, 3] and known to mediate neuroinflammation[4, 5]. A similar assumption can be made on Celf4 which has important roles in post-transcriptional regulation of gene expression[6]. Moreover, a deeper understanding can be gained through more focused spatiotemporal analysis. For example, when we inspect module 48, which includes Alcam, we can observe other addiction behavior-related genes such as Gabra4[7, 8] Jak2[9], etc. Its expression profile provides a more detailed insight into altering behavior in spatiotemporal context, showing orchestrated decreasing pattern from mid timepoint (S10 Fig).

In the PFC, a region responsible for executive functions such as decision-making and impulse control, cocaine exposure leads to alterations in the expression of genes involved in synaptic plasticity and neurotransmitter signaling[10-13]. These changes may contribute to the cognitive deficits and impaired decision-making often observed in individuals with cocaine addiction. In our study, both modules 16 and 17 are annotated with the primary GO term of regulation of dendritic spine morphogenesis.

We observe that the expression profile of module 16 displays a consistent increase in expression since the beginning of cocaine self-administration, which is distinct from the spatiotemporal expression profile of module 48, even both modules have a high correlation with addictive behavior (S10 Fig).

Lastly, the VTA, a primary source of dopaminergic projections to the NAc and PFC, undergoes changes that can modulate the activity of dopaminergic neurons and influence the development of addiction [14]. Module 10, which is related to addictive behavior and annotated with the primary GO term of retinoic acid metabolic process, not only aligns with previous studies suggesting retinoic acid as a possible contributor to affective disorders[15, 16], but also provides unique insights in the spatiotemporal aspect. This is evident in its expression profile, which displays a temporal decrease and recovery pattern in the mid-timepoint (S10 Fig).

Module 10, which is related to addictive behavior and annotated with the primary GO term of retinoic acid metabolic process, not only aligns with previous studies suggesting retinoic acid as a possible contributor to affective disorders (24, 25), but also provides unique insights in the spatiotemporal aspect. This is evident in its expression profile, which displays a temporal decrease and recovery pattern in the mid-timepoint (S10 Fig)

Reference

1. Scofield MD, Heinsbroek JA, Gipson CD, Kupchik YM, Spencer S, Smith ACW, et al. The Nucleus Accumbens: Mechanisms of Addiction across Drug Classes Reflect the Importance of Glutamate Homeostasis. Pharmacological Reviews. 2016;68(3):816-71. doi: 10.1124/pr.116.012484.
2. Lyck R, Lécuyer M-A, Abadier M, Wyss CB, Matti C, Rosito M, et al. ALCAM (CD166) is involved in extravasation of monocytes rather than T cells across the blood–brain barrier. Journal of Cerebral Blood Flow & Metabolism. 2016;37(8):2894-909. doi: 10.1177/0271678X16678639.

3. Verma A, Bennett J, Örme AM, Polycarpou E, Rooney B. Cocaine addicted to cytoskeletal change and a fibrosis high. Cytoskeleton. 2019;76(2):177-85. doi: https://doi.org/10.1002/cm.21510.
4. Sil S, Niu F, Tom E, Liao K, Periyasamy P, Buch S. Cocaine Mediated Neuroinflammation: Role of Dysregulated Autophagy in Pericytes. Mol Neurobiol. 2019;56(5):3576-90. Epub 2018/08/29. doi: 10.1007/s12035-018-1325-0. PubMed PMID: 30151726; PubMed Central PMCID: PMC6393223.
5. Kohno M, Link J, Dennis LE, McCready H, Huckans M, Hoffman WF, et al. Neuroinflammation in addiction: A review of neuroimaging studies and potential immunotherapies. Pharmacol Biochem Behav. 2019;179:34-42. Epub 2019/01/30. doi: 10.1016/j.pbb.2019.01.007. PubMed PMID: 30695700; PubMed Central PMCID: PMC6637953.
6. Wagnon JL, Briese M, Sun W, Mahaffey CL, Curk T, Rot G, et al. CELF4 Regulates Translation and Local Abundance of a Vast Set of mRNAs, Including Genes Associated with Regulation of Synaptic Function. PLOS Genetics. 2012;8(11):e1003067. doi: 10.1371/journal.pgen.1003067.
7. Levran O, Peles E, Randesi M, Correa da Rosa J, Ott J, Rotrosen J, et al. Glutamatergic and GABAergic susceptibility loci for heroin and cocaine addiction in subjects of African and European ancestry. Prog Neuropsychopharmacol Biol Psychiatry. 2016;64:118-23. Epub 2015/08/19. doi: 10.1016/j.pnpbp.2015.08.003. PubMed PMID: 26277529; PubMed Central PMCID: PMC4564302.
8. Stephens DN, King SL, Lambert JJ, Belelli D, Duka T. GABAA receptor subtype involvement in addictive behaviour. Genes, Brain and Behavior. 2017;16(1):149-84. doi: https://doi.org/10.1111/gbb.12321.
9. Berhow MT, Hiroi N, Kobierski LA, Hyman SE, Nestler EJ. Influence of cocaine on the JAK-STAT pathway in the mesolimbic dopamine system. J Neurosci. 1996;16(24):8019-26. Epub 1996/12/15. doi: 10.1523/jneurosci.16-24-08019.1996. PubMed PMID: 8987828; PubMed Central PMCID: PMC6579209.
10. Goldstein RZ, Volkow ND. Dysfunction of the prefrontal cortex in addiction: neuroimaging findings and clinical implications. Nat Rev Neurosci. 2011;12(11):652-69. Epub 2011/10/21. doi: 10.1038/nrn3119. PubMed PMID: 22011681; PubMed Central PMCID: PMC3462342.
11. Bolla K, Ernst M, Kiehl K, Mouratidis M, Eldreth D, Contoreggi C, et al. Prefrontal cortical dysfunction in abstinent cocaine abusers. J Neuropsychiatry Clin Neurosci. 2004;16(4):456-64. Epub 2004/12/24. doi: 10.1176/jnp.16.4.456. PubMed PMID: 15616172; PubMed Central PMCID: PMC2771441.
12. Kawahara Y, Ohnishi YN, Ohnishi YH, Kawahara H, Nishi A. Distinct Role of Dopamine in the PFC and NAc During Exposure to Cocaine-Associated Cues. Int J Neuropsychopharmacol. 2021;24(12):988-1001. Epub 2021/10/10. doi: 10.1093/ijnp/pyab067. PubMed PMID: 34626116; PubMed Central PMCID: PMC8653875.
13. Otis JM, Mueller D. Reversal of Cocaine-Associated Synaptic Plasticity in Medial Prefrontal Cortex Parallels Elimination of Memory Retrieval. Neuropsychopharmacology. 2017;42(10):2000-10. doi: 10.1038/npp.2017.90.
14. Yang H, de Jong JW, Tak Y, Peck J, Bateup HS, Lammel S. Nucleus Accumbens Subnuclei Regulate Motivated Behavior via Direct Inhibition and Disinhibition of VTA Dopamine Subpopulations. Neuron. 2018;97(2):434-49.e4. Epub 2018/01/09. doi: 10.1016/j.neuron.2017.12.022. PubMed PMID: 29307710; PubMed Central PMCID: PMC5773387.
15. Oliva I, Wanat MJ. Ventral Tegmental Area Afferents and Drug-Dependent Behaviors. Front Psychiatry. 2016;7:30. Epub 2016/03/26. doi: 10.3389/fpsyt.2016.00030. PubMed PMID: 27014097; PubMed Central PMCID: PMC4780106.
16. Bremner JD, McCaffery P. The neurobiology of retinoic acid in affective disorders. Prog Neuropsychopharmacol Biol Psychiatry. 2008;32(2):315-31. Epub 2007/08/21. doi: 10.1016/j.pnpbp.2007.07.001. PubMed PMID: 17707566; PubMed Central PMCID: PMC2704911.
