# Supplementary material for: GAN-WGCNA: Calculating gene modules to identify key intermediate regulators in cocaine addiction
Source: PLoS One. 2024 Oct 3;19(10):e0311164. doi: 10.1371/journal.pone.0311164 (PMC11449371; doi:10.1371/journal.pone.0311164)
Supplement: S5 File — (PDF) [file pone.0311164.s018.pdf]

S5 Note. Summary and comparison between WGCNA and GAN-WGCNA

Detecting co-expressed gene modules and calculating correlation between gene modules and traits using WGCNA are well-accepted analytic method. In these analytic methods, a trait, which can be numeric or categorical value, is compared with eigengene of a module. The eigengene represents the expression profile of a co-expressed module, allowing conclusions to be drawn, such as a high expression level of module corresponding to a high trait value. Indeed, including this study which calculated about addictive behavior(the highest module has a 0.82 correlation in VTA; Table S1), several studies already showed the effectiveness of correlation analysis in neuroscience domain such as PFC for sex(-0.14 to 0.24 of correlation)[1], PFC for age(-0.67 to 0.56 of correlation)[1], alcohol addiction(-0.28 to 0.15 of correlation)[2], etc. which help to predict causality between gene expression levels and traits.

In this study, we followed a similar process but modified intermediate steps of module detection and correlation calculation. Unlike conventional WGCNA, which calculates eigengenes from their real samples that also used in module detection, GAN-WGCNA employs time-series generated samples for module detection and real samples for module-trait correlation analysis.

Detecting modules based on time-series generated samples and calculating eigengenes from real samples requires extra effort but offers several advantages. GAN-WGCNA is able to provide module-trait correlation information which is not just quantitative proportional information between trait and gene level, but similarity information in temporal aspect based on time-series levels of gene and trait, because the module itself defined in time-series expression level and has a trait value in its real sample based eigengene simultaneously, therefore (S11 Fig). Indeed, this improvement provides more detailed assistance in our manuscript and supplementary notes compared to conventional WGCNA.

References

1. Hu Y, Pan J, Xin Y, Mi X, Wang J, Gao Q, et al. Gene Expression Analysis Reveals Novel Gene Signatures Between Young and Old Adults in Human Prefrontal Cortex. Front Aging Neurosci. 2018;10:259. Epub 2018/09/14. doi: 10.3389/fnagi.2018.00259. PubMed PMID: 30210331; PubMed Central PMCID: PMC6119720.

2. Kapoor M, Wang J-C, Farris SP, Liu Y, McClintick J, Gupta I, et al. Analysis of whole genome-transcriptomic organization in brain to identify genes associated with alcoholism. Translational Psychiatry. 2019;9(1):89. doi: 10.1038/s41398-019-0384-y.
