# Supplementary material for: GAN-WGCNA: Calculating gene modules to identify key intermediate regulators in cocaine addiction
Source: PLoS One. 2024 Oct 3;19(10):e0311164. doi: 10.1371/journal.pone.0311164 (PMC11449371; doi:10.1371/journal.pone.0311164)
Supplement: Attachment — Submitted filename: Response to Reviewers.docx [file pone.0311164.s019.docx]

**Reviewer 1**

The manuscript presents a novel analytical pipeline combining Generative Adversarial Networks (GAN) with Weighted Gene Co-expression Network Analysis (WGCNA) to identify key regulatory genes involved in cocaine addiction. Utilizing time-series gene expression data from C57BL/6J mice subjected to two weeks of cocaine self-administration, the study leverages GAN to generate intermediate transcriptome profiles, enabling detailed analysis of gene module interactions over time. Key findings include the identification of two significant genes, Alcam and Celf4, which show high correlation with addictive behavior. The study demonstrates the utility of GAN-WGCNA in uncovering hidden intermediate regulators, providing valuable insights into the spatiotemporal dynamics of gene expression in cocaine addiction.

Here are five technical or conceptual questions based on the provided manuscript titled "GAN-WGCNA: calculating gene modules as a way to find key intermediate regulators in cocaine addiction":

**Comment 1**: How does the integration of Generative Adversarial Networks (GAN) with Weighted Gene Co-expression Network Analysis (WGCNA) enhance the identification of key gene modules involved in cocaine addiction compared to traditional methods?

**Response 1**: Traditional methods for calculating gene modules require datasets from different samples. Moreover, identifying gene modules that include temporal information necessitates time-series sample acquisition from experimental animals. Given that addiction is not only a time-series process but also a result of the interplay between multiple brain regions, the difficulties of sample acquisition are significantly higher than in other research subjects.

GAN-WGCNA provides a time-series gene expression profile without the need for intermediate samples and calculates its correlation with external traits. We demonstrated that by applying GAN-WGCNA to a previously published bulk dataset, which did not include intermediate samples, we could identify spatiotemporal gene modules and genes that were missed in previous studies but were consistent with known literature. The correlation metrics obtained were highly accepted in conventional biological research. These comprehensive and well acceptable results stem from the integration of GAN and traditional WGCNA methods.

**Comment 2**: What are the specific biological implications of the identified intermediate genes (Alcam and Celf4) in the context of cocaine addiction, and how might these genes influence addiction-related behaviors and neural processes?

**Response 2**: We were unable to deliver conclusive results on the roles of Alcam and Celf4 in cocaine addiction; addressing this question requires larger-scale research, including molecular, structural, and experimental studies. However, we can emphasize the biological implications within the context of GAN-WGCNA. Both Alcam and Celf4 were missed in previous studies, yet they have known correlations with addiction. Alcam is involved in cell-cell adhesion, which is crucial for synaptic plasticity and neural connectivity, potentially influencing the neural circuits involved in addiction. Celf4 regulates RNA processing and local translation, which are critical for synaptic function and plasticity. These findings suggest that GAN-WGCNA is capable of capturing intermediate process from bulk dataset in both intercellular and intracellular phenomena. For better presentation for those significance and implications, we updated our manuscript as follows.

“

We proposed generative adversarial networks and weighted gene co-expression network analysis (GAN-WGCNA) pipeline and rescued differentially expressed gene(rDEG) methods to provide an analysis for intermediate regulators. GAN can be used to generate time-series gene expression profiles without any intermediate sample collection between control and pathological state. WGCNA is an un-biased systemic level analysis method which suitable to analyze generated time-series gene expression profiles. Combination of these two methods, GAN-WGCNA indeed provides information about time-series interplay of gene modules during cocaine addiction. Moreover, rDEG rescued two intermediate regulators (Alcam and Celf4) of cocaine addiction which were missed in previous research. Considering that Alcam and Celf4 have known correlations with addiction-related behaviors. Alcam is involved in cell-cell adhesion, which is crucial for synaptic plasticity and neural connectivity, potentially influencing the neural circuits involved in addiction. Celf4 regulates RNA processing and local translation, which are critical for synaptic function and plasticity. These findings suggest that GAN-WGCNA is capable of capturing intermediate process from bulk dataset in both intercellular and intracellular phenomena.

In summary, understanding intermediate regulators in spatiotemporal pathogenesis is essential to treatment and individual components of this pipeline are already well accepted methods and provide qualitative and quantitative windows to biological researchers, presented work will contribute to various future research and drug development.

”

**Comment 3**: Can you elaborate on the methodology used to ensure the statistical significance and biological relevance of the generated intermediate gene expression profiles using the GAN approach?

**Comment 4**: What challenges did the researchers encounter when aligning temporal gene expression profiles with cocaine addiction stages, and how were these challenges addressed in the study?

**Response 3 and 4**: In this research, we acknowledge that our approach using GAN may not yet be fully accepted by the biological research community. This is why we validate our results with known facts and literature and chose to use the same equations for calculating statistical significance and biological relevance that are used in conventional WGCNA and DEG analyses. By adhering to these well-established methods, we aim to ensure the credibility and acceptance of our findings. Therefore, elaborating on the equations for calculating statistical significance or biological relevance methods from the aspect of GAN alone may miss the intentions of this research. We may address these elaborations in a subsequent study.

**Comment 5**: How does the rDEG (rescued differentially expressed gene) method contribute to the identification of intermediate regulatory genes, and what are the advantages of this approach over traditional DEG analysis in the context of cocaine addiction research?

**Response 5**: In the study, intermediate key genes are first filtered from intermediate gene modules that are correlated with behavioral traits. Even though their statistical significance of correlation meets the conventional WGCNA criteria, there are still about a hundred genes in each module, and their individual quantitative significance is not considered. The rDEG (rescued differentially expressed gene) method contributes by using the generated time-series profiles from GAN to calculate DEGs across the dataset. This approach ensures that the statistical significance of each gene is validated in a well-accepted manner. By employing rDEG, we are able to confirm the quantitative significance of the genes within these modules, providing a more robust and precise analysis compared to traditional DEG methods. This validation process enhances the reliability of identifying intermediate regulatory genes in the context of cocaine addiction research.

**Comment:** Here are the grammatical and phrasing errors identified in the provided manuscript along with corrections.

Title and Abstract

1. Title:

- Error: "GAN-WGCNA: calculating gene modules as a way to find key intermediate regulators in cocaine addiction"

- Correction: "GAN-WGCNA: Calculating Gene Modules to Identify Key Intermediate Regulators in Cocaine Addiction"

2. Abstract, Line 3:

- Error: "Spatio-temporally enriched NGS data contain important underlying regulatory machinery of biological processes."

- Correction: "Spatio-temporally enriched NGS data contain important underlying regulatory mechanisms of biological processes."

3. Abstract, Line 4-5:

- Error: "Generative adversarial network (GAN) has been used to augment biological data so that it can describe hidden intermediate time-series gene expression profiles during specific biological processes."

- Correction: "Generative adversarial networks (GANs) have been used to augment biological data to describe hidden intermediate time-series gene expression profiles during specific biological processes."

4. Abstract, Line 6-7:

- Error: "The development of a pipeline that uses an augmented time-series gene expression profile is needed to provide an unbiased systemic level map of biological processes and tests for statistical significance of the generated dataset, which leads to discovery of hidden intermediate regulator."

- Correction: "Developing a pipeline that uses augmented time-series gene expression profiles is needed to provide an unbiased systemic-level map of biological processes and test for the statistical significance of the generated dataset, leading to the discovery of hidden intermediate regulators."

Introduction

1. Introduction, Line 1-2:

- Error: "Complex time-series interplay of genes can be observed in various biological processes, such as the cell cycle(1-3), circadian rhythm(4, 5), development(6), and pathogenesis."

- Correction: "Complex time-series gene interactions can be observed in various biological processes, such as the cell cycle (1-3), circadian rhythm (4, 5), development (6), and pathogenesis."

2. Introduction, Line 6-7:

- Error: "Functionality of key factors at specific spatiotemporal points – where, when, and how–is important to this process."

- Correction: "The functionality of key factors at specific spatiotemporal points—where, when, and how—is important to this process."

3. Introduction, Line 9:

- Error: "Typical single-cell RNA sequencing data have 20–30,000 cells with over 10,000 genes."

- Correction: "Typical single-cell RNA sequencing data consist of 20,000–30,000 cells with over 10,000 genes."

4. Introduction, Line 13:

- Error: "Although the network is spatiotemporally complex, in most cases, the current pipeline provides only weak or no spatiotemporal regulatory information."

- Correction: "Although the network is spatiotemporally complex, in most cases, the current pipeline provides only weak or no spatiotemporal regulatory information."

Methods

1. Methods, Line 2:

- Error: "Detailed method is described in Supplementary note 1."

- Correction: "The detailed method is described in Supplementary Note 1."

2. Methods, Line 4:

- Error: "Detailed method is described in Supplementary note 2."

- Correction: "The detailed method is described in Supplementary Note 2."

3. Methods, Line 6:

- Error: "For example, we used the RMSprop optimizer instead of the AdamOptimizer."

- Correction: "For example, we used the RMSprop optimizer instead of the Adam Optimizer."

4. Methods, Line 8:

- Error: "This process was repeated until a successful resembled fake generation was obtained using different NumPy seed values."

- Correction: "This process was repeated until a successful resembled fake generation was obtained using different NumPy seed values."

Results

1. Results, Line 1:

- Error: "In a previous study (18), transcriptome-wide regulation was evaluated in six reward-associated brain regions (PFC, DStr;CPU, NAc, BLA, vHIP, VTA) by comparing DEG analysis in different contexts (acute, re-exposure, etc.) and combining the behavioral addiction index."

- Correction: "In a previous study (18), transcriptome-wide regulation was evaluated in six reward-associated brain regions (PFC, DStr, CPU, NAc, BLA, vHIP, VTA) by comparing DEG analysis in different contexts (acute, re-exposure, etc.) and combining the behavioral addiction index."

2. Results, Line 3:

- Error: "The GAN method uses bulk mRNA-seq data as the training input and generates a time-series intermediate transcriptome using latent space interpolation."

- Correction: "The GAN method uses bulk mRNA-seq data as the training input and generates a time-series intermediate transcriptome using latent space interpolation."

3. Results, Line 5:

- Error: "The generated gene expression profile provided enriched spatiotemporal information."

- Correction: "The generated gene expression profile provided enriched spatiotemporal information."

4. Results, Line 7:

- Error: "The expression profile of the Creb family shows diverse properties of spatiotemporal gene expression changes, revealing subfamily specific profile patterns."

- Correction: "The expression profile of the Creb family shows diverse properties of spatiotemporal gene expression changes, revealing subfamily-specific profile patterns."

Discussion

1. Discussion, Line 1:

- Error: "Machine learning is a promising computational method that can be used to overcome existing limitations in many domains, including biological research (42)."

- Correction: "Machine learning is a promising computational method that can be used to overcome existing limitations in many domains, including biological research (42)."

2. Discussion, Line 4:

- Error: "We proposed a GAN-WGCNA method using generated gene expression data, which is an unbiased systemic approach for the full utilization of high temporal resolution."

- Correction: "We proposed a GAN-WGCNA method using generated gene expression data, which is an unbiased systemic approach for the full utilization of high temporal resolution."

3. Discussion, Line 6:

- Error: "These approaches showed that gene modules in the NAc are mostly involved in addiction processes which account for previous studies (16, 26, 44, 45)."

- Correction: "These approaches showed that gene modules in the NAc are mostly involved in addiction processes, consistent with previous studies (16, 26, 44, 45)."

4. Discussion, Line 10:

- Error: "Most genes belong to the very first or last time points because their expression profile simply decreases or increases, making it meaningless to some extent."

- Correction: "Most genes belong to the very first or last time points because their expression profiles simply decrease or increase, making it meaningless to some extent."

5. Discussion, Line 12:

- Error: "An alternative algorithm or principle is required for generated data that have a higher resolution or dimensionality compared to traditional experimental data."

- Correction: "An alternative algorithm or principle is required for generated data that have a higher resolution or dimensionality compared to traditional experimental data."

**Response**: Thanks for comment, although some grammatical errors may appear ambiguous, we followed your corrections except the “Results, Line 1” which Dstr was also denoted CPU in original study.

**Reviewer 2**

The authors presents a study for enhancing the resolution and analysis of time-series gene expression data, leading to the identification of critical genes involved in cocaine addiction. The combination of GAN and WGCNA represents a significant advancement in the field of bioinformatics and addiction research. I would like to recommend this manuscript for publication after the authors address/answer the following comments.

**Comment 1:** In the introduction section, the authors briefly described the two methods employed in this study. For the GAN-WGCNA, which is the combination of the GAN and WGCNA methods, it would be beneficial for the reader to understand the importance by adding more details, such as how these two models are correlated.

**Response 1**: We appreciate the comment and have added more details in the introduction section to explain how GAN and WGCNA are correlated as below.

“

Accompanying with GAN, Weighted Gene Co-expression Network Analysis (WGCNA) was used in this study which is a powerful systemic biology method used to describe the correlation patterns among genes across multiple samples. This method calculates a network based on gene expression data, where nodes represent genes and edges reflect the strength of the correlation between gene pairs. By grouping genes with similar expression patterns into modules, WGCNA allows researchers to identify clusters of co-expressed genes that may share common biological functions or regulatory mechanisms. WGCNA is one of well-accepted conventional methods that particularly valuable for exploring complex biological processes and diseases, as it integrates high-dimensional gene expression data to study gene networks associated with specific phenotypes or conditions.

”

**Comment 2:** In the “Wasserstein Generative Adversarial Networks with Gradient Penalty Loss” section, the authors explained the modifications made to the previous WGAN-GP model and achieved a Pearson correlation value of 0.95. Have you tried further modifying the model to increase the correlation value closer to 1?

**Response 2**: We acknowledge the importance of achieving higher accuracy. Further modifications to increase the correlation value were considered, but we focused on maintaining a balance between model complexity, computational efficiency, and generalization. Given that our resembled fakes are built from real samples and subsequently averaged into gene profiles, efforts to further increase accuracy would be suboptimal. However, we plan to explore additional model optimizations in future work to achieve even higher correlation values. It is also worth noting that our criteria were slightly more stringent than those used in previous studies.

**Comment 3:** Could you update some figures, such as Figure 2, 4b, to a higher resolution in order to clearly present the data points?

**Response 3**: As you requested, we updated the figure’s resolution as follows.

**Comments 4**: In the conclusion section, the authors present the Alcam and Celf4 as the miss cocaine addiction gene in the previous study. It would be advantageous to revisit the biological significance of these genes to underscore their relevance in the context of this study.

**Response 4**: We’d like to emphasize that both Alcam and Celf4 were missed in previous studies, yet they have known correlations with addiction. Alcam and Celf4 have known correlations with addiction-related behaviors. Alcam is involved in cell-cell adhesion, which is crucial for synaptic plasticity and neural connectivity, potentially influencing the neural circuits involved in addiction. Celf4 regulates RNA processing and local translation, which are critical for synaptic function and plasticity. These findings suggest that GAN-WGCNA is capable of capturing intermediate process from bulk dataset in both intercellular and intracellular phenomena. We updated those statement of significance in conclusion section as follows.

“

We proposed generative adversarial networks and weighted gene co-expression network analysis (GAN-WGCNA) pipeline and rescued differentially expressed gene(rDEG) methods to provide an analysis for intermediate regulators. GAN can be used to generate time-series gene expression profiles without any intermediate sample collection between control and pathological state. WGCNA is an un-biased systemic level analysis method which suitable to analyze generated time-series gene expression profiles. Combination of these two methods, GAN-WGCNA indeed provides information about time-series interplay of gene modules during cocaine addiction. Moreover, rDEG rescued two intermediate regulators (Alcam and Celf4) of cocaine addiction which were missed in previous research. Considering that Alcam and Celf4 have known correlations with addiction-related behaviors. Alcam is involved in cell-cell adhesion, which is crucial for synaptic plasticity and neural connectivity, potentially influencing the neural circuits involved in addiction. Celf4 regulates RNA processing and local translation, which are critical for synaptic function and plasticity. These findings suggest that GAN-WGCNA is capable of capturing intermediate process from bulk dataset in both intercellular and intracellular phenomena.

In summary, understanding intermediate regulators in spatiotemporal pathogenesis is essential to treatment and individual components of this pipeline are already well accepted methods and provide qualitative and quantitative windows to biological researchers, presented work will contribute to various future research and drug development.

”

**Reviewer 3**

The paper titled "GAN-WGCNA: calculating gene modules as a way to find key intermediate regulators in cocaine addiction" introduces an approach to decipher the time-series interactions of genes, which is critical for understanding the diagnosis and treatment of diseases. Specifically, this study focuses on cocaine addiction. The authors developed a pipeline that leverages Generative Adversarial Networks (GAN) to enhance biological data, paired with Weighted Gene Co-expression Network Analysis (WGCNA), to identify hidden intermediate regulators during biological processes.

In their analysis, the authors examined a transcriptome dataset from a two-week cocaine self-administration experiment in C57BL/6J mice. Through the GAN-WGCNA method, they identified two genes, Alcam and Celf4, as significant intermediaries correlated with addiction behavior. The study emphasizes that these genes showed notable statistical significance in their correlation with addiction behavior, and their expression and co-regulation were mapped across time, brain regions, and biological processes.

I think this idea is interesting. However, there are several concerns that lead me to recommend rejecting this paper. These concerns include technical novelty, lack of good comparison and validation experiments, insufficient description of methods, writing issues, and potential concerns about their idea.

**Comment 1**: Method Paper Clarity

If this is intended to be a method paper, the abstract and introduction should be rewritten to better reflect that. The abstract should start by mentioning the key gap in current techniques. Additionally, validating your method in at least two datasets would strengthen your claims, as currently, only one is used. It is also important to highlight the novelty of this approach more clearly.

**Response 1**: We appreciate the comment and have revised the abstract and introduction to better reflect the methodological focus of the paper. We have emphasized the key gap in current techniques and highlighted the novelty of our approach. However, addressing your concern regarding the use of additional datasets for validation may be beyond the scope of this research.

There are several reasons for this. First, in terms of concrete validation of the generated time-series profiles, equivalent sample generation at each time point is required, which is beyond our current capacity or scope. Second, broad cross-validation of using GAN in research has already been addressed by previous studies employing GANs. We are also working on addressing more biological problems using our framework, which makes it difficult to handle this aspect in the current study.

We plan to incorporate additional datasets and further validations in future research to strengthen our findings, and we look forward to publishing these results in future studies.

“

To understand the spatiotemporal complexity, several experimental methods have been devised. One experi-mental approach is systems biology, particularly -omics data generation using sequencing technology. Typical single-cell RNA sequencing (scRNA-seq) data, which reads gene expression levels of each cell, consist of 20,000–30,000 cells with over 10,000 genes. Meanwhile, bulk RNA sequencing reads gene expression levels of each tissue sample, usually up to a few hundred. Basic interpretation can be done using Differentially Expressed Gene (DEG) calculations from bulk RNA sequencing, which typically identify over one hundred significant genes. In most biological studies using these hundred components, research should involve complex gene regulatory machinery. Although the network is spatiotemporally complex, the current pipeline in most cases provides only weak or no spatiotemporal regulatory information.

”

**Comment 2**: Literature Review

In the introduction, the statement that LASSO isn’t optimal for feature reduction is understandable. However, LASSO is a fundamental method, and its concept is different from GAN-WGCNA. It would be beneficial to cite more papers with similar ideas to handle such situations. How do other researchers manage the lack of data samples when doing similar work? More comprehensive literature review for this specific part would be helpful. While the next paragraph cites several papers using GAN for augmenting biological data, it is crucial to explain why GAN is particularly suitable for this context.

**Response 2**: We have expanded the literature review in the introduction to include more citations of similar methodologies and approaches that address the challenge of limited data samples. We also provided a more detailed explanation of why GAN is particularly suitable for this context, emphasizing its ability to generate realistic intermediate states that enhance temporal resolution.

Additionally, we are developing more powerful and advanced methods evolved from our current framework, including GAN. We look forward to publishing these results in future studies.

“

To overcome or compensate for expensive experimental methods, several in silico approaches have been devised. For example, Marouf et al. demonstrated that generating real-like omics data with non-linear properties is possible using advanced computational techniques[1]. However, simply generating more data may not be sufficient for credibility and adding insights to previous studies. Instead, unveiling a hidden trajectory of the transcriptome between the initial and final phases of experimental conditions affected by disease or drugs with finer time resolution could provide a better lens to observe minute details of pathogenesis-related gene modules without any additional experiments.

”

**Comment 3**: Methods

The method section would benefit from more details on how GAN was used to augment the data.

(1) Providing necessary mathematical formulations and explaining how the model was trained would be valuable.

(2) It is also important to address how you ensured that the model is not overfitting, especially if a training-test split was not used.

(3) Some evidence supporting the choice of GAN for modeling gene expression would also strengthen the manuscript. Given the fact GAN is notorious difficult to train all the times.

**Response 3**:

**Response 3-(1):** We have added more details on the mathematical formulations and training process of the GAN model in the methods section.

“

The Gulrajani, et al suggested following loss for training.

$$\min_{G} \max_{D\in\mathcal{D}} E_{x\sim\mathbb{P}_{r}}\left[ D\left( x \right) \right]-E_{\tilde{x}\sim\mathbb{P}_{g}}\left[ D\left( \tilde{x} \right) \right]$$

$$L=E_{\tilde{x}\sim\mathbb{P}_{g}}\left[ D\left( \tilde{x} \right) \right]-E_{x\sim\mathbb{P}_{r}}\left[ D\left( x \right) \right]+\lambda E_{\hat{x}\sim\mathbb{P}_{\hat{x}}}\left[ \left( ||\nabla_{\hat{x}}D\left( \hat{x} \right)||_{2}-1 \right)^{2} \right]$$

”

**Response 3-(2):** We first observed the discriminator's performance during training to check whether it performs exceptionally well on the training data but poorly on the validation data. Second, we monitored the losses of both the generator and the discriminator during training. Lastly, we visually inspected the generated dataset to ensure it well-covered the original dataset in tSNE plot, including test data.

**Response 3-(3):** There are several reasons we chose GAN. First, the idea of using GAN to generate intermediate transcriptomes is neither entirely new nor fully accepted, but it has been explored in a few publications. Second, GANs have the advantage of generating more realistic data compared to other methods. Lastly, we are developing more powerful and advanced methods evolved from our current framework, including GAN, and have conducted over 100 trainings using different biological dataset. We look forward to publishing these results in future studies.

**Comment 4**: Conceptually I disagree with this paper

The paper claims that GAN is powerful for augmenting data points, which is true. However, GAN is typically used to generate data points that are as identical to the original as possible. It would be helpful to clarify how this method can be used to understand the time-series interplay of genes for diagnosis and treatment of disease. Explaining how the generated data points contribute to the analysis would address potential concerns. For example, won’t a better method to be variational autoencoder? By playing with the key element in the hidden states you can actually have some artificial datapoints in the middle of two categories, which might mimic the time interplay better.

**Response 4**: We appreciate the conceptual feedback. The generated datapoints consist of time-series expression profiles for each gene, which offers two key advantages. First, the increased dimensionality allows for more detailed gene module detection with conventional WGCNA. Second, these time-series profiles enable analysis under a temporal aspect. By calculating the correlation between gene modules and behavioral traits using time-series detected gene modules, we can describe the time-series interplays correlated with behavior. If we were to apply WGCNA on raw bulk datasets alone, we would only obtain sparsely grained gene modules and eigengene profile which consisted by gene expression level across samples which lack the temporal information therefore unable to use to describe the time-series interplay of brain regions.

As you suggest, Variational autoencoders (VAEs) are indeed another powerful method, we chose GANs for their superior performance in generating high-fidelity intermediate states and other reasons related our latest research. We acknowledge the potential advantages of VAEs and recognize them as a promising direction for future research.

**Comment 5**: Results Section:

The statement "GAN enables a calculation of correlation between gene module and behavioral data” needs more clarity. It would be useful to explain why the original dataset does not support similar analysis and whether other data augmentation methods could achieve similar results. Highlighting how your method differs from existing approaches, or the original data would be beneficial. The manuscript should clearly show the additional information or insights provided by your method.

**Response 5**: As described in the manuscript and in Response 4, the generated dataset allows for a more dedicated basis for temporal analysis. The key idea is to calculate gene modules in the generated time-series gene expression profile and then recalculate their eigen gene profiles in real samples, which may significantly contribute to addressing the correlation problems. Moreover, in the original dataset, the intermediate expression levels observed over the 2-week addiction process do not exist without GAN-mediated generation in bulk transcriptome analysis. While other data augmentation methods might have similar potential to contribute, we have not yet identified any that offer the same advantages in terms of dataset, methods, and analysis of results. We have added this point in our manuscript as follows.

“

We proposed a GAN-WGCNA method using generated gene expression data, which is an unbiased systemic approach for the full utilization of high temporal resolution. Our method, which combines GAN and WGCNA, improved its analytic results and enabled not only unbiased analysis but also calculation of the correlation between temporal gene modules and addiction behavior. The key idea of combining GAN and WGCNA is to detect gene modules in the generated time-series profile of each gene and calculate their eigen gene profiles in real samples that have trait information. Indeed, these approaches showed that gene modules in the NAc are mostly involved in addiction processes, consistent with previous studies [2-5]. We rescued DEGs in the intermediate stages of cocaine addiction using the rDEG method. This enables the understanding of time-series biological processes. GAN-WGCNA combined with rDEG, has the capability of unbiased and intermediate-level analysis, that allowed the rescue of at least two important genes in the intermediate stage. – Celf4 [6, 7], and Alcam [8].

”

**Comment 6**: Suggested Experiment: A useful experiment could be using a large dataset and applying your GAN-WGCNA method on a subset of that data. Checking if you can find differentially expressed genes (DEG) or GO pathways identifiable only in the larger cohort but not in the smaller subset could validate the method’s effectiveness.

**Response 6**: Indeed, we agree that large scale experiment with sample acquisition equivalent to the generated dataset would strengthen our credibility. However, obtaining samples at each time point is beyond our current capacity or scope. Therefore, this will be addressed in future studies.

**Comment 7**: Result Section Organization: Some result sections are quite long and may be confusing for readers. Consider restructuring them to make the manuscript more organized and easier to follow.

**Response 7**: We appreciate the feedback regarding the organization of the results section. To make the manuscript more organized and easier to follow, we have added statements to each paragraph to help clarify our arguments and improve readability. We believe these changes will enhance the overall structure and flow of the results section, making it more accessible for readers.

“

In summary, we enabled qualitative screening of intermediate key gene modules and genes that show correla-tion with addictive behavior, both cross-checked in literature and validated in a well-accepted manner using convention-al WGCNA methods. This approach ensures the robustness and relevance of our findings in the context of spatiotem-poral study including addiction research

…

In summary, we enabled quantitative measurement of intermediate key genes identified in previous analyses by applying conventional Differentially Expressed Gene (DEG) methods to the generated time-series gene expression profiles. This approach allowed us to validate the significance of these genes in a dynamic context, providing a more detailed and robust understanding of their roles in well-acceptable manner.

“

**Reviewer 4**

With this manuscript titled “GAN-WGCNA: calculating gene modules as a way to find key intermediate regulators in cocaine addiction” the authors analyzed (1) a transcriptome dataset of two weeks of cocaine self-administration in C57BL/6J mice. (2) found two genes (Alcam and Celf4) as intermediate significant genes that showed high correlation with cocaine addiction behavior via GAN-WGCNA analysis method.

The manuscript is pleasant to read. But I still have some concerns that need to be addressed.

**Comment 1**: Since the author mentioned the raw data is from previous literature, I read the previous paper and found they have data from six groups mice for cocaine self-administration. The current manuscript results based on all 6 groups or limited groups?

**Response 1**: We trained our dataset using all six groups and conditions from the previous study. However, the time-series profiles were generated only for the cocaine self-administration, as it describes the transcriptome changes specifically associated with cocaine addiction.

**Comment 2**: The original paper (Ref.18) found cocaine addiction genes highly related with basolateral amygdala (BLA), but the current manuscript showed different results (Compare BLA vs. NAC). How to explain it?

**Response 2**: Our methods are designed to rescue missed information from conventional analyses, which may result in conclusions that partially align with previous studies while also uncovering additional information. The differences observed in our results compared to the original paper highlight the additional insights gained through our approach. We would also like to note that our findings are consistent with other research in the field, further validating the robustness of our method.

**Comment 3**: Cocaine withdraw data is also important, it will be good to check if Alcam and Celf4 genes are also related to cocaine short time (24 hr) or long time (30 d) withdraw with GAN-WGCNA since the ref.18 had found some interesting results from the short and long withdraw groups.

**Response 3**: We appreciate the suggestion and recognize the importance of studying cocaine withdrawal data. Indeed, we are open to conducting follow-up studies not only using our code and dataset but also providing the raw data of cocaine withdrawal data upon request. We plan to cover these questions in future research to provide a more comprehensive understanding of the roles these genes play during different stages of cocaine withdrawal.

1.	Marouf M, Machart P, Bansal V, Kilian C, Magruder DS, Krebs CF, et al. Realistic in silico generation and augmentation of single-cell RNA-seq data using generative adversarial networks. Nature Communications. 2020;11(1):166. doi: 10.1038/s41467-019-14018-z.

2.	Teague CD, Nestler EJ. Key transcription factors mediating cocaine-induced plasticity in the nucleus accumbens. Molecular Psychiatry. 2021. doi: 10.1038/s41380-021-01163-5.

3.	Pierce RC, Fant B, Swinford-Jackson SE, Heller EA, Berrettini WH, Wimmer ME. Environmental, genetic and epigenetic contributions to cocaine addiction. Neuropsychopharmacology. 2018;43(7):1471-80. doi: 10.1038/s41386-018-0008-x.

4.	Kourrich S, Rothwell PE, Klug JR, Thomas MJ. Cocaine Experience Controls Bidirectional Synaptic Plasticity in the Nucleus Accumbens. The Journal of Neuroscience. 2007;27(30):7921-8. doi: 10.1523/jneurosci.1859-07.2007.

5.	Scofield MD, Heinsbroek JA, Gipson CD, Kupchik YM, Spencer S, Smith ACW, et al. The Nucleus Accumbens: Mechanisms of Addiction across Drug Classes Reflect the Importance of Glutamate Homeostasis. Pharmacological Reviews. 2016;68(3):816-71. doi: 10.1124/pr.116.012484.

6.	Wagnon JL, Briese M, Sun W, Mahaffey CL, Curk T, Rot G, et al. CELF4 Regulates Translation and Local Abundance of a Vast Set of mRNAs, Including Genes Associated with Regulation of Synaptic Function. PLOS Genetics. 2012;8(11):e1003067. doi: 10.1371/journal.pgen.1003067.

7.	Bryant CD, Yazdani N. RNA-binding proteins, neural development and the addictions. Genes, Brain and Behavior. 2016;15(1):169-86. doi: <https://doi.org/10.1111/gbb.12273>.

8.	Bye CR, Rytova V, Alsanie WF, Parish CL, Thompson LH. Axonal Growth of Midbrain Dopamine Neurons is Modulated by the Cell Adhesion Molecule ALCAM Through <em>Trans</em>-Heterophilic Interactions with L1cam, Chl1, and Semaphorins. The Journal of Neuroscience. 2019;39(34):6656-67. doi: 10.1523/jneurosci.0278-19.2019.
