# Supplementary material for: GAN-WGCNA: Calculating gene modules to identify key intermediate regulators in cocaine addiction
Source: PLoS One. 2024 Oct 3;19(10):e0311164. doi: 10.1371/journal.pone.0311164 (PMC11449371; doi:10.1371/journal.pone.0311164)
Supplement: Attachment — Submitted filename: Response to Reviewers.docx [file pone.0311164.s020.docx]

**Reviewer 2**

The authors present a study for enhancing the resolution and analysis of time-series gene expression data, leading to the identification of critical genes involved in cocaine addiction. After the revision, I would like to recommend this manuscript after the authors address/answer the following comment.

**Comment 1**: the authors added the extra paragraph to explain the correlation between GAN and WGCNA methods. Although the extra detailed paragraph really helped to understand the relationship between two models, it will be beneficial to including the reference for several statement, such as “WGCNA is one of well-accepted conventional methods that….”

**Response 1**: In response to the reviewer’s comment, we have added references to support the statements and note that references are updated accordingly.

“

Generative Adversarial Network (GAN) may be a solution for this problem. A GAN has been used in several domains, including image processing, not only to achieve a higher performance generative model with a lesser amount of training data [1-5],

…

WGCNA is one of well-accepted conventional methods that particularly valuable for exploring complex biological processes and diseases, as it integrates high-dimensional gene expression data to study gene networks associated with specific phenotypes or conditions [6-10].

”

1.	Goodfellow I, Pouget-Abadie J, Mirza M, Xu B, Warde-Farley D, Ozair S, et al. Generative adversarial nets. Advances in neural information processing systems. 2014;27.

2.	Radford A. Unsupervised representation learning with deep convolutional generative adversarial networks. arXiv preprint arXiv:151106434. 2015.

3.	Lan Z, Zhou B, Zhao W, Wang S. An optimized GAN method based on the Que-Attn and contrastive learning for underwater image enhancement. PLOS ONE. 2023;18(1):e0279945. doi: 10.1371/journal.pone.0279945.

4.	Shen Y, Huang R, Huang W. GD-StarGAN: Multi-domain image-to-image translation in garment design. PLOS ONE. 2020;15(4):e0231719. doi: 10.1371/journal.pone.0231719.

5.	Bouchard C, Wiesner T, Deschênes A, Bilodeau A, Turcotte B, Gagné C, et al. Resolution enhancement with a task-assisted GAN to guide optical nanoscopy image analysis and acquisition. Nature Machine Intelligence. 2023;5(8):830-44. doi: 10.1038/s42256-023-00689-3.

6.	Feltrin ASA, Tahira AC, Simões SN, Brentani H, Martins DC, Jr. Assessment of complementarity of WGCNA and NERI results for identification of modules associated to schizophrenia spectrum disorders. PLOS ONE. 2019;14(1):e0210431. doi: 10.1371/journal.pone.0210431.

7.	DiLeo MV, Strahan GD, den Bakker M, Hoekenga OA. Weighted Correlation Network Analysis (WGCNA) Applied to the Tomato Fruit Metabolome. PLOS ONE. 2011;6(10):e26683. doi: 10.1371/journal.pone.0026683.

8.	Wen B, Chen J, Ding T, Mao Z, Jin R, Wang Y, et al. Development and experimental validation of hypoxia-related gene signatures for osteosarcoma diagnosis and prognosis based on WGCNA and machine learning. Scientific Reports. 2024;14(1):18734. doi: 10.1038/s41598-024-69638-3.

9.	Sun Q, Wang Z, Xiu H, He N, Liu M, Yin L. Identification of candidate biomarkers for GBM based on WGCNA. Scientific Reports. 2024;14(1):10692. doi: 10.1038/s41598-024-61515-3.

10.	Renz PF, Ghoshdastider U, Baghai Sain S, Valdivia-Francia F, Khandekar A, Ormiston M, et al. In vivo single-cell CRISPR uncovers distinct TNF programmes in tumour evolution. Nature. 2024;632(8024):419-28. doi: 10.1038/s41586-024-07663-y.
